# Supplementary material for: Circadian Expression of Migratory Factors Establishes Lineage-Specific Signatures that Guide the Homing of Leukocyte Subsets to Tissues
Source: Immunity. 2018 Dec 18;49(6):1175–1190.e7. doi: 10.1016/j.immuni.2018.10.007 (PMC6303219; doi:10.1016/j.immuni.2018.10.007)
Supplement: Document S1. Figures S1–S7 [file mmc1.pdf]

Immunity, Volume *49*

Supplemental Information

Circadian Expression of Migratory Factors Establishes Lineage-Specific Signatures that Guide the Homing of Leukocyte Subsets to Tissues

Wenyan He, Stephan Holtkamp, Sophia Martina Hergenhan, Kerstin Kraus, Alba de Juan, Jasmin Weber, Paul Bradfield, Julien Martin Pierre Grenier, Jeoffrey Pelletier, David Druzd, Chien-Sin Chen, Louise Madeleine Ince, Susanne Bierschenk, Robert Pick, Markus Sperandio, Michel Aurrand-Lions, and Christoph Scheiermann

Figure S1

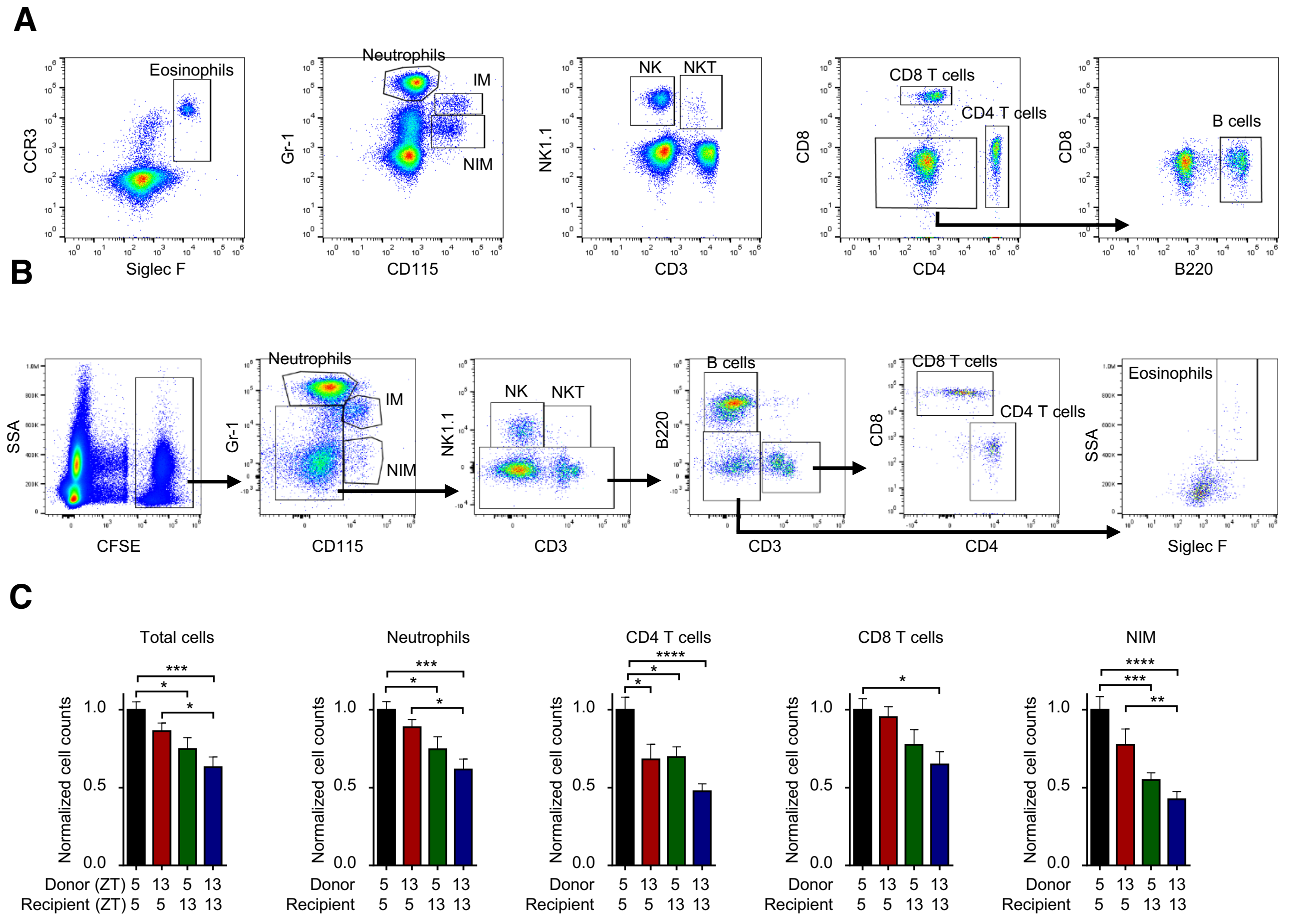

<u>Figure S1 (Related to Figure 1) *Gating strategies and reciprocal homing assays*</u>

(A) Gating strategy of blood leukocyte subsets for Figure 1A. **(B)** Gating strategy for identifying adoptively transferred CFSE^{+} leukocyte subsets in recipient blood. **(C)** Reciprocal 'negative' homing assays with ZT5 and ZT13 donor cells labeled differently and co-injected into ZT5 and ZT13 recipients; n = 21-24 mice, one-way ANOVA followed by Tukey's multiple comparison test. *p<0.05, **p<0.01, ***p<0.001, ****p<0.0001.

Figure S2

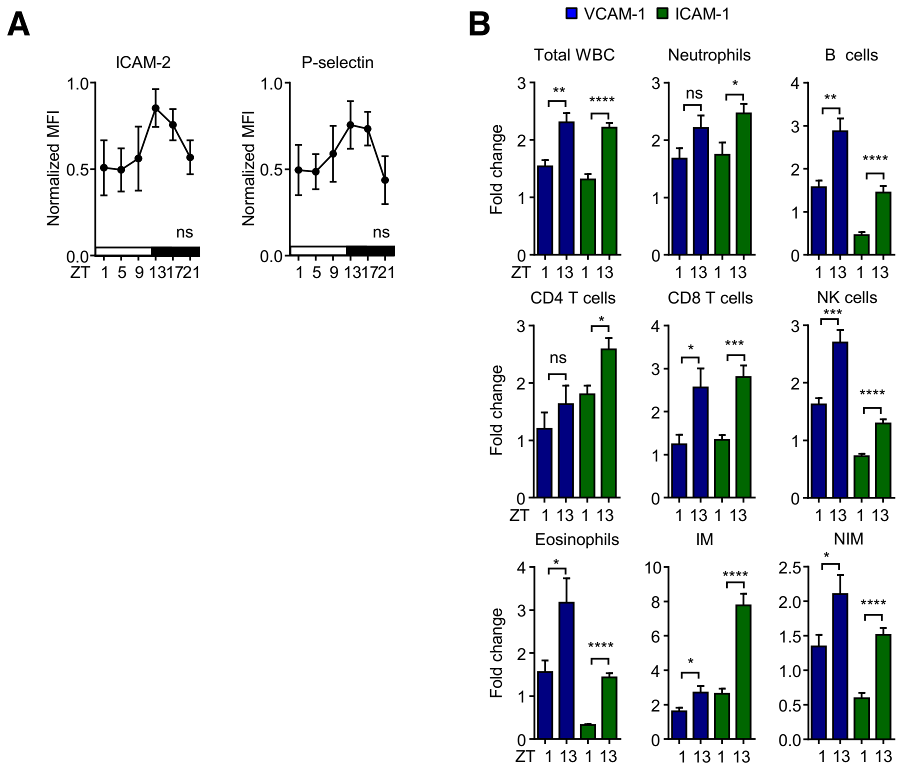

<u>Figure S2 (Related to Figure 2) *Oscillations in endothelial cell adhesion molecules and associated functions*</u>

(A) Integration of ICAM-2 and P-selectin expression over all organs across the day; n = 3-6 mice with 6 time points measured each, one-way ANOVA. **(B)** Fold change of endogenous leukocyte numbers after treatment with antibodies directed against VCAM-1 or ICAM-1 compared to ZT1 and ZT13 isotype-treated control groups; n = 7-11 mice, unpaired Student's t-test. *$p<0.05$, **$p<0.01$, ***$p<0.001$, ****$p<0.0001$. ns, not significant.

Figure S3

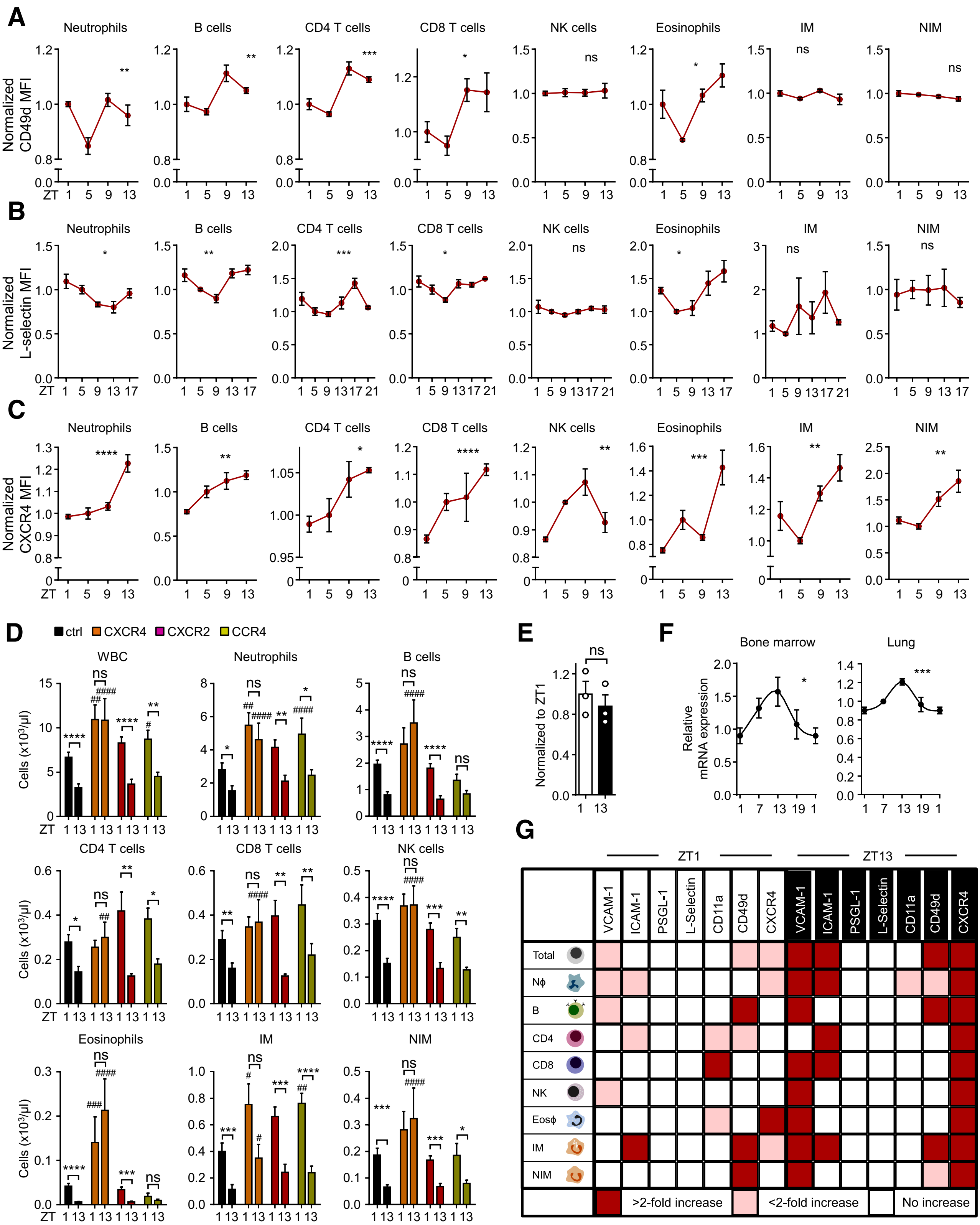
A
Neutrophils
B cells
CD4 T cells
CD8 T cells
NK cells
Eosinophils
IM
NIM
Normalized CD49d MFI
ZT
B
Normalized L-selectin MFI
C
Normalized CXCR4 MFI
D
ctrl
CXCR4
CXCR2
CCR4
WBC
Cells (x10³/µl)
E
Normalized to ZT1
F
Bone marrow
Lung
Relative mRNA expression
G
ZT1
ZT13
VCAM-1
ICAM-1
PSGL-1
L-Selectin
CD11a
CD49d
CXCR4
Total
Nϕ
B
CD4
CD8
NK
Eosϕ
IM
NIM
>2-fold increase
<2-fold increase
No increase

<u>Figure S3 (Related to Figure 3)</u> ***<u>Oscillations in leukocyte pro-migratory factors and associated functions</u>***

(A-C) Expression levels of CD49d **(A)**, L-selectin **(B)** and CXCR4 **(C)** on leukocyte subsets over 24h; n = 3-6 mice with 4-6 time points measured each, one-way ANOVA. **(D)** Endogenous blood leukocyte numbers after treatment with chemokine receptor antagonists; n = 5-12 mice, one-way ANOVA followed by Dunnett comparison to control groups. **(E)** Adoptive transfer of donor cells treated *ex vivo* prior to transfer to recipients with antagonists against CXCR4 at ZT1 and ZT13. Data are normalized to ZT1 levels; n = 3 mice, unpaired Student's t-test. **(F)** *Cxcl12* mRNA levels in bone marrow and lung, ZT1 is double plotted to facilitate viewing; n = 4-5 mice, one-way ANOVA. **(G)** Overview of functional blocking effects on endogenous leukocyte subsets in blood targeting the indicated molecules at ZT1 and ZT13; n = 4-12 mice, one-way ANOVA followed by Dunnett comparison to control groups. *p<0.05, **p<0.01, ***p<0.001, ****p<0.0001; $^{\#,\ \#\#,\ \#\#\#,\ \#\#\#\#}$ indicate analogous significance levels to controls. ns, not significant.

Figure S4

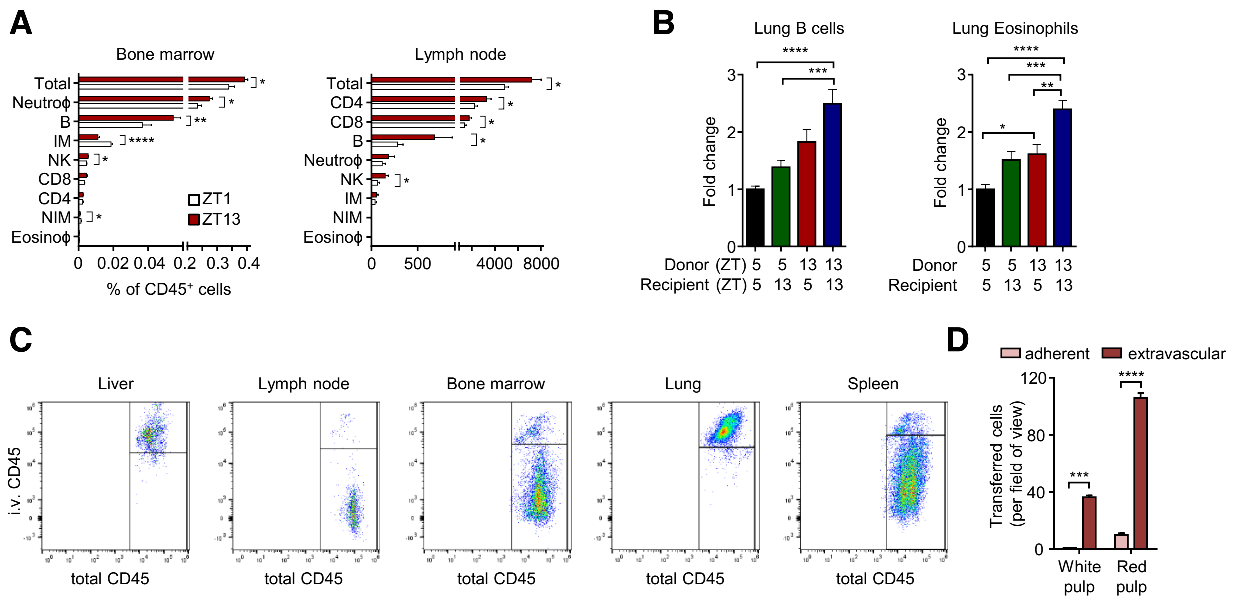

Figure S4 (Related to Figure 4) *Diurnal homing capacity of leukocyte subsets and localization in specific organs*

(A) Recruitment of donor cells into bone marrow and lymph nodes with ZT1 and ZT13 donor cells and recipients; bone marrow, n = 24-25 mice; lymph node, n = 11-14 mice, unpaired Student's t-test. **(B)** Reciprocal homing assays with ZT5 and ZT13 donor cells labeled differently and co-injected into ZT5 and ZT13 recipients; n = 10 mice, one-way ANOVA followed by Tukey's multiple comparison test. **(C)** Flow cytometry plots of adoptively transferred cells located inside or outside the vasculature in specific organs based on staining with an i.v.-injected anti-CD45 antibody. **(D)** Quantification of numbers and localization of adoptively transferred cells in splenic white pulp and red pulp; n = 2-3 mice, unpaired Student's t-test. *p<0.05, **p<0.01, ***p<0.001, ****p<0.0001.

Figure S5

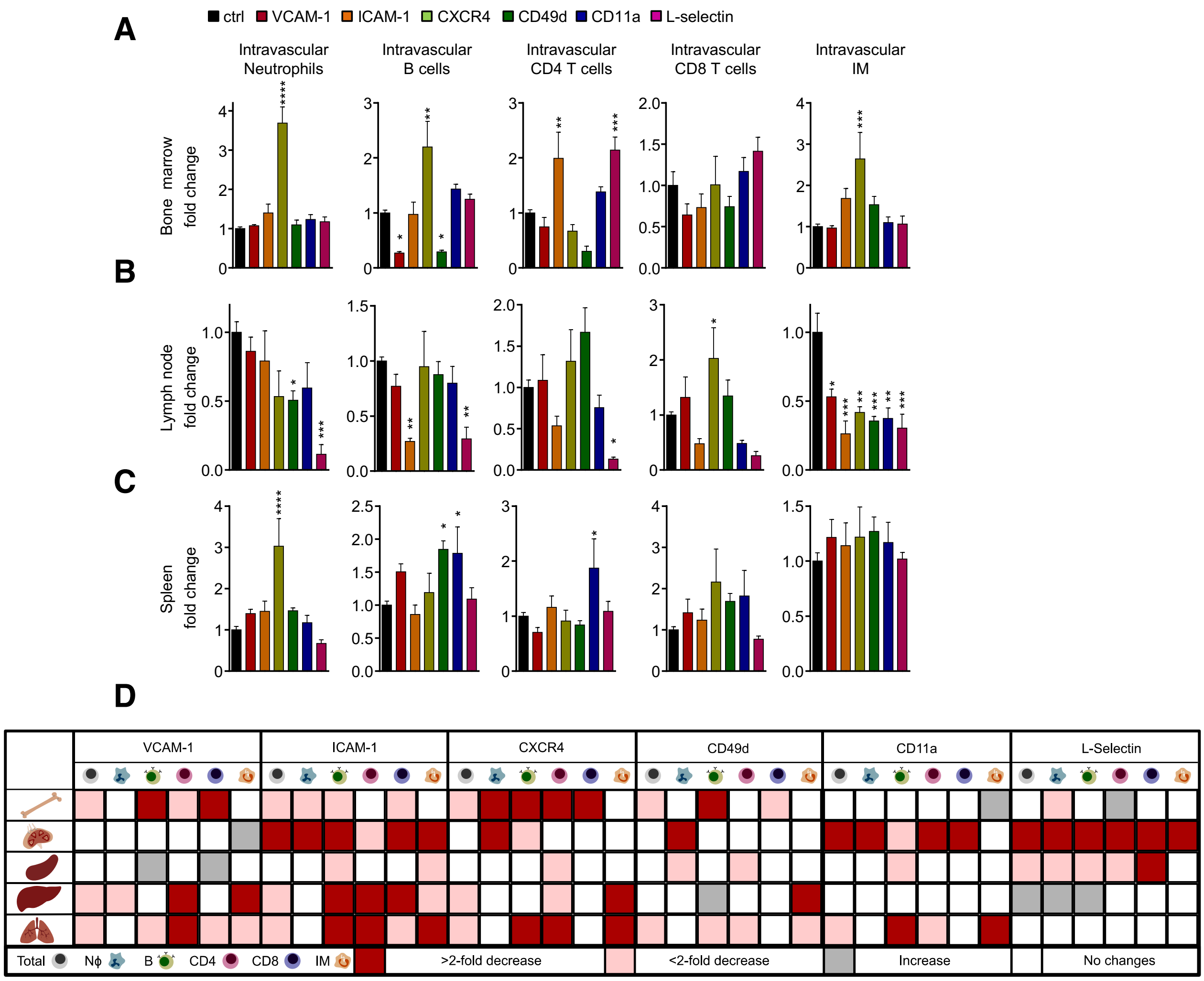

<u>Figure S5 (Related to Figure 5) *Chronopharmacological targeting of leukocyte homing to tissues*</u>

(A-C) Numbers of intravascular donor cells after adoptive transfer and treatment with antibodies or antagonists directed against the indicated molecules, n = 4-8 mice, one-way ANOVA followed by Dunnett comparison to the control group. **(D)** Overview of functional blocking effects on leukocyte recruitment to organs targeting the indicated molecules, n = 4-8 mice, one-way ANOVA followed by Dunnett comparison to the control group. *p<0.05, **p<0.01, ***p<0.001, ****p<0.0001.

Figure S6

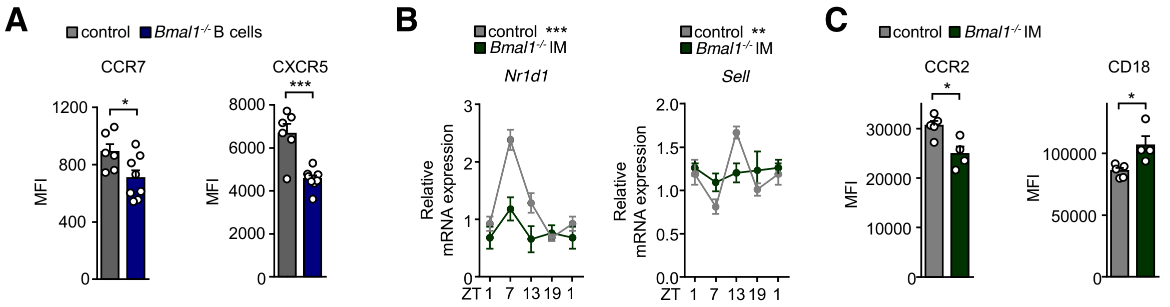

Figure S6 (Related to Figure 6) *Expression of leukocyte adhesion molecules in lineage-specific Bmal1$^{-/-}$ leukocytes*

(A) Expression levels of CCR7 and CXCR5 at ZT13 in control and *Bmal1*-deficient B cells in blood; n = 6-8 mice, unpaired Student's t-test. **(B)** Q-PCR analyses of *Nr1d1* and *Sell* mRNA levels in isolated control and *Bmal1*-deficient monocytes; n = 3 mice, one-way ANOVA. **(C)** Expression levels of CCR2 and CD18 at ZT13 in control and *Bmal1*-deficient monocytes in blood; n = 4-5 mice, unpaired Student's t-test. *p<0.05, **p<0.01, ***p<0.001, ****p<0.0001.

Figure S7

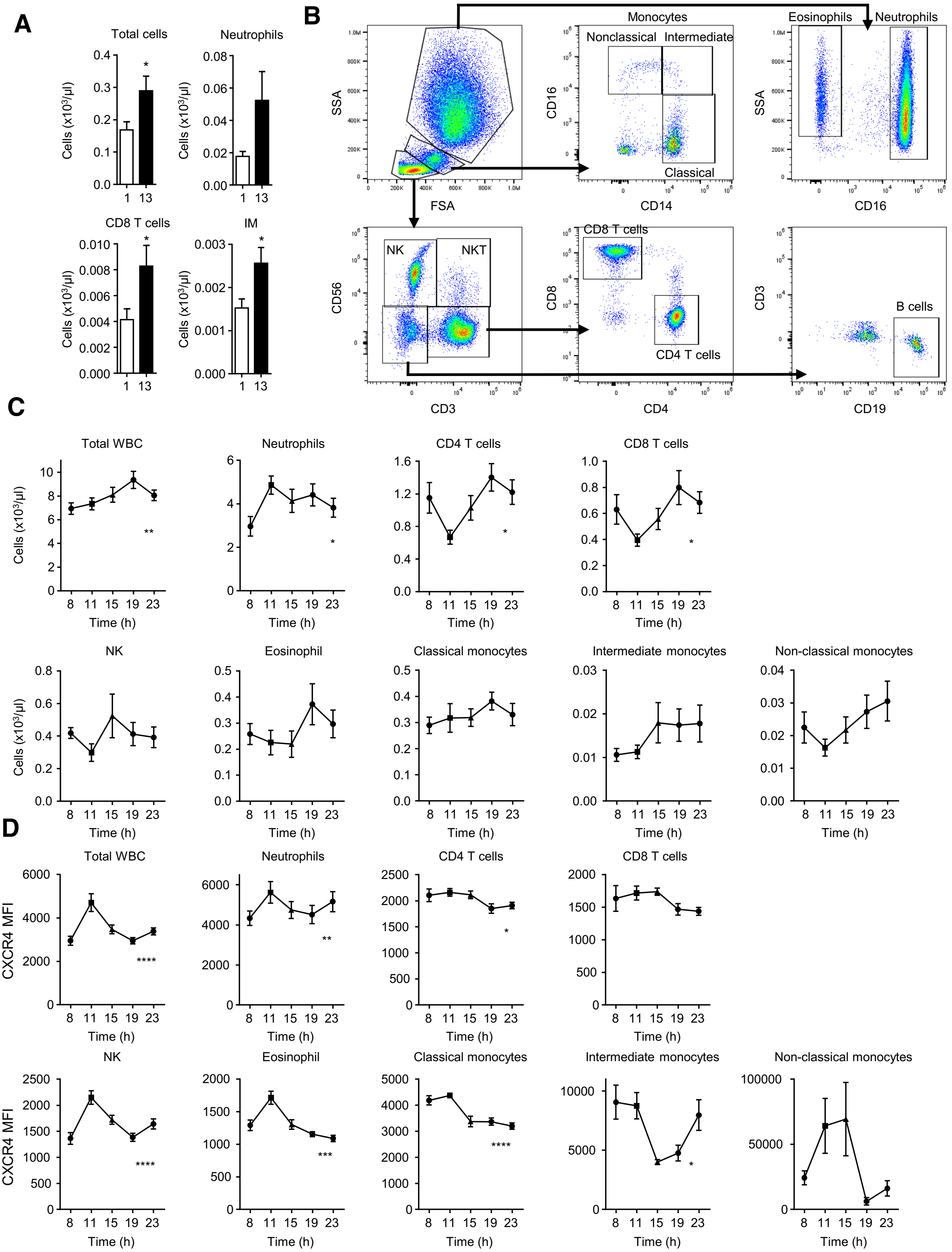
A
Total cells
Neutrophils
CD8 T cells
IM
Cells (x10³/µl)
1 13
B
SSA
FSA
Monocytes
Nonclassical
Intermediate
Classical
CD16
CD14
Eosinophils
Neutrophils
NK
NKT
CD56
CD3
CD8 T cells
CD4 T cells
CD8
CD4
B cells
CD19
C
Total WBC
Neutrophils
CD4 T cells
CD8 T cells
NK
Eosinophil
Classical monocytes
Intermediate monocytes
Non-classical monocytes
Time (h)
D
CXCR4 MFI

Figure S7 (Related to Figure 7) ***Oscillations in inflammation and human leukocytes in blood***

(A) Numbers of total leukocytes and subsets in the peritoneal cavity after i.p. LPS challenge at ZT1 and ZT13; n = 12-14 mice, unpaired Student's t-test. **(B)** Gating strategy of human blood leukocyte subsets. **(C)** Numbers of human blood leukocyte subsets over 24h; n = 8 subjects, repeated measures one-way ANOVA. **(D)** Expression of CXCR4 on human leukocytes over 24h; n = 8 subjects, repeated measures one-way ANOVA. *p<0.05, **p<0.01, ***p<0.001, ****p<0.0001.
