# Supplementary material for: Circadian Expression of Migratory Factors Establishes Lineage-Specific Signatures that Guide the Homing of Leukocyte Subsets to Tissues
Source: Immunity. 2018 Dec 18;49(6):1175–1190.e7. doi: 10.1016/j.immuni.2018.10.007 (PMC6303219; doi:10.1016/j.immuni.2018.10.007)
Supplement: Document S2. Article plus Supplemental Information [file mmc4.pdf]

Resource

Immunity

Circadian Expression of Migratory Factors Establishes Lineage-Specific Signatures that Guide the Homing of Leukocyte Subsets to Tissues

Graphical Abstract

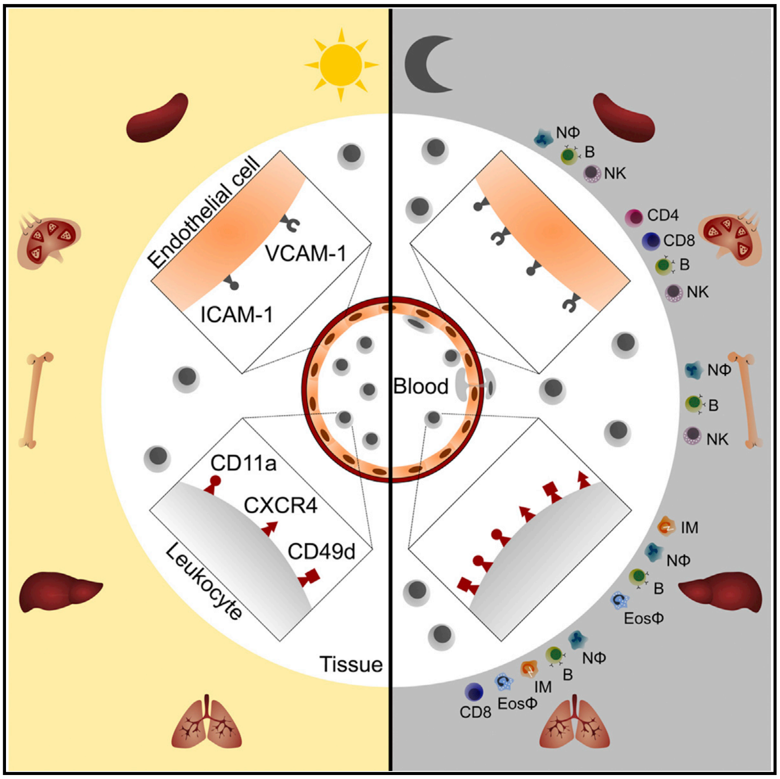

Highlights

- Leukocyte subsets show time-of-day-dependent migration patterns to organs
- This relies on lineage- and tissue-specific oscillations in pro-migratory factors
- Loss of circadian clocks in the endothelium or leukocytes ablates rhythmicity
- The efficacy of blocking leukocyte migration is time-of-day dependent

Authors

Wenyan He, Stephan Holtkamp, Sophia Martina Hergenhan, ..., Markus Sperandio, Michel Aurrand-Lions, Christoph Scheiermann

Correspondence

christoph.scheiermann@med.uni-muenchen.de

In Brief

Leukocytes continuously circulate throughout the body. He et al. demonstrate that trafficking patterns of major leukocyte subsets occur in a rhythmic manner dependent on the time-of-day-dependent expression of lineage- and tissue-specific factors. This influences the inflammatory response and leukemic tumor burden and translates to the migration behavior of human primary lymphocytes.

He et al., 2018, Immunity 49, 1175–1190
December 18, 2018 © 2018 The Author(s). Published by Elsevier Inc.
https://doi.org/10.1016/j.immuni.2018.10.007

CellPress

CellPress

Circadian Expression of Migratory Factors Establishes Lineage-Specific Signatures that Guide the Homing of Leukocyte Subsets to Tissues

Wenyan He,[1] Stephan Holtkamp,[1] Sophia Martina Hergenhan,[1] Kerstin Kraus,[1] Alba de Juan,[1] Jasmin Weber,[1] Paul Bradfield,[2] Julien Martin Pierre Grenier,[3] Jeoffrey Pelletier,[3] David Druzd,[1] Chien-Sin Chen,[1] Louise Madeleine Ince,[1,5] Susanne Bierschenk,[1] Robert Pick,[1] Markus Sperandio,[1,4] Michel Aurrand-Lions,[3] and Christoph Scheiermann[1,4,5,6,*]

[1]Walter Brendel Centre of Experimental Medicine, University Hospital, Ludwig Maximilians University of Munich, BioMedical Centre, 82152 Planegg-Martinsried, Germany
[2]Mesenflow Technologies SARL, Fondation Eclosion, Geneva, Switzerland
[3]Aix-Marseille University, Centre National de la Recherche Scientifique, INSERM, Institut Paoli-Calmettes, Centre de Recherche en Cancérologie de Marseille, Marseille, France
[4]DZHK (German Centre for Cardiovascular Research), partner site Munich Heart Alliance, Munich, Germany
[5]Department of Pathology and Immunology, Centre Médical Universitaire, University of Geneva, Switzerland
[6]Lead Contact
*Correspondence: christoph.scheiermann@med.uni-muenchen.de
https://doi.org/10.1016/j.immuni.2018.10.007

SUMMARY

The number of leukocytes present in circulation varies throughout the day, reflecting bone marrow output and emigration from blood into tissues. Using an organism-wide circadian screening approach, we detected oscillations in pro-migratory factors that were distinct for specific vascular beds and individual leukocyte subsets. This rhythmic molecular signature governed time-of-day-dependent homing behavior of leukocyte subsets to specific organs. Ablation of BMAL1, a transcription factor central to circadian clock function, in endothelial cells or leukocyte subsets demonstrated that rhythmic recruitment is dependent on both microenvironmental and cell-autonomous oscillations. These oscillatory patterns defined leukocyte trafficking in both homeostasis and inflammation and determined detectable tumor burden in blood cancer models. Rhythms in the expression of pro-migratory factors and migration capacities were preserved in human primary leukocytes. The definition of spatial and temporal expression profiles of pro-migratory factors guiding leukocyte migration patterns to organs provides a resource for the further study of the impact of circadian rhythms in immunity.

INTRODUCTION

Leukocytes exit the blood by undergoing extensive interactions with endothelial cells. This sequence of events is known as the leukocyte adhesion cascade (Butcher, 1991; Ley et al., 2007; Muller, 2016; Springer, 1994; Vestweber, 2015; Wagner and Frenette, 2008). Circulating leukocytes first tether along endothelial cells by engaging P-selectin glycoprotein ligand-1 (PSGL-1) with E- and P-selectin presented on the endothelium. This process brings the cells in closer proximity to the vessel wall and slows them down to roll along endothelial cells, using PSGL-1 as well as L-selectin to interact with endothelial selectins. During this step, leukocytes come in contact with chemokines presented on the endothelial cell surface. Chemokines engage chemokine receptors on leukocytes, leading to Gαi-mediated inside-out signaling of integrins. Integrins extend into a high-affinity conformation and mediate the firm adhesion of leukocytes. Lymphocyte function-associated antigen-1 (LFA-1) (CD11a/CD18 or αLβ2 integrin), macrophage-1 antigen (Mac-1) (CD11b/CD18 or αMβ2 integrin), and very late antigen-4 (VLA-4) (CD49d/CD29 or α4β1 integrin) play a major role in this step and interact with members of the immunoglobulin superfamily on endothelial cells, primarily intercellular adhesion molecule-1 (ICAM-1), ICAM-2, and vascular cell adhesion molecule-1 (VCAM-1). In the final step, adherent leukocytes crawl along the vessel wall, probe for adequate sites for crossing the endothelium, and emigrate from the vascular lumen into the parenchyma in a process termed transmigration.

The requirement of specific molecules in the leukocyte emigration process is highly dependent on the tissue context and the leukocyte subset involved (Schnoor et al., 2015). Interactions between receptor-ligand pairs of pro-migratory molecules governing the subset-specific migration process of leukocytes to specific organs have been referred to as a homing code (Marelli-Berg et al., 2008; Rot and von Andrian, 2004; Springer, 1994). Although some of these molecular binding partners are known and have been discussed above, the trafficking requirements of many leukocyte subsets are unclear. This is particularly true for the steady state because leukocyte infiltration into organs has mostly been studied in inflammatory scenarios.

Recent data point to the influence of time of day on the number of leukocytes present in the circulation (Casanova-Acebes et al., 2013; Druzd et al., 2017; Nguyen et al., 2013; Scheiermann et al., 2012; Shimba et al., 2018; Suzuki et al., 2016). These circadian rhythms, occurring within a period of approximately 24 h, are

This is an open access article under the CC BY license (http://creativecommons.org/licenses/by/4.0/).

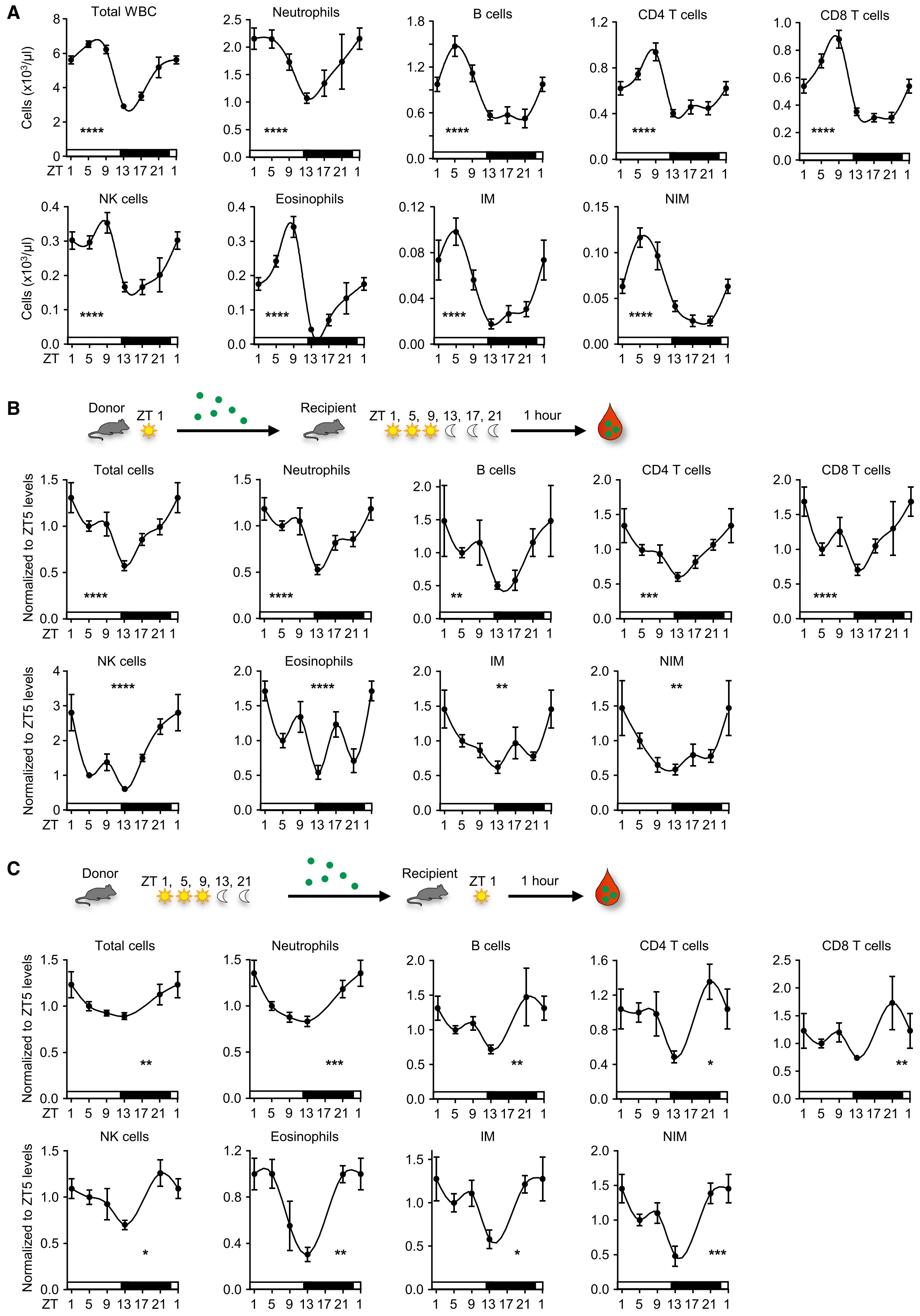

(legend on next page)

critical in aligning the body to the usual recurring cycles of the environment (Arjona et al., 2012; Curtis et al., 2014; Dibner et al., 2010; Labrecque and Cermakian, 2015; Man et al., 2016; Scheiermann et al., 2018; Scheiermann et al., 2013). The number of leukocytes circulating in blood is largely dependent on two factors: mobilization into blood from organs such as bone marrow, which increases cellularity in blood (input); and emigration from blood into organs, decreasing cellularity in blood (output). Here, we investigated the hypothesis that leukocyte subsets migrate to organs at specific times of the day. By employing this diurnal rhythmicity as a functional screening tool in combination with a systematic approach of adoptive transfer and homing assays, we detected a time-resolved code of pro-migratory factors for the specific migration behavior of leukocyte subsets to organs. Lineage-specific genetic ablation of the circadian clock demonstrated that endothelial-cell- and leukocyte-autonomous oscillations are critical in these processes. These rhythms are relevant in inflammation and determine leukemic tumor burden at specific times. Human primary leukocytes exhibit a similar time-resolved code of pro-migratory factors. The circadian patterns of expression of pro-migratory factors defined here present a resource for the further exploration of how the immune system has adapted to the recurring cycle of the environment and to the relevance of this adaptation in health and disease.

RESULTS

Rhythmic Emigration of Leukocyte Subsets from Blood

Circulating white blood cell (WBC) counts oscillate in murine blood such that they exhibit a peak 5 hr after the onset of light (also known as Zeitgeber time [ZT] 5, i.e., 5 hr after lights on [12 p.m.] in a 12 hr/12 hr light/dark environment) and a trough in the evening (ZT13 or 8 p.m., 1 hr after lights off) (Figure 1A). Numbers of neutrophils, B cells, CD4 and CD8 T cells, natural killer (NK) cells, NK T cells, eosinophils, and inflammatory and non-inflammatory monocytes showed similar peaks and troughs with a 2- to 7-fold change in numbers between the peak and trough depending on the subset (Figure 1A, Figure S1A, and data not shown). We investigated whether a rhythmic leukocyte emigration process could explain the observed oscillations in blood. As an initial screen, we performed ‘‘negative’’ homing assays, where 1 hr after adoptively transferring leukocytes intravenously (i.v.), we quantified the number of transferred cells remaining in the blood to assess emigration of cells across the whole organism. To additionally investigate the influence of a rhythmic microenvironment in this process, we harvested donor cells at one time and transferred them simultaneously into recipients that were kept in shifted light cycles. We saw a clear diurnal rhythm given that the number of labeled donor cells remaining in the blood after transfer was lowest in an evening environment (ZT13) and highest in the morning (ZT1) for all investigated subsets (Figure 1B and Figure S1B). This demonstrated that in the evening more cells had left the blood, for example, by migrating into tissues or by firm contact with the vasculature, both of which effectively removed them from the circulation. Furthermore, it demonstrated a strong influence of rhythmicity in the microenvironment on leukocyte recruitment and numbers in blood in general. We next assessed the role of rhythmicity in leukocytes in this process. This time, donor cells were harvested from mice kept in shifted light cycles and simultaneously injected into recipient mice at one time of the day. In this scenario as well, ‘‘evening’’ cells showed the highest emigration behavior, and ‘‘morning’’ cells generally showed the lowest (Figure 1C). We confirmed these observations by performing reciprocal emigration assays where ‘‘morning’’ or ‘‘evening’’ cells were co-injected into ‘‘morning’’ or ‘‘evening’’ recipients, respectively, with differential color labeling (Figure S1C). These data thus demonstrated that both microenvironment and leukocytes co-contribute to rhythmic leukocyte exit from the circulation, a broad phenomenon that peaks in the evening for all subsets investigated.

Tissue-Specific Oscillations in Endothelial Cell Adhesion Molecules

Because the microenvironment is a strong driver of rhythmic leukocyte emigration from blood (Figure 1B), we performed a screen of multiple organs for oscillatory expression of adhesion molecules on endothelial cells, the initial points of contacts for leukocytes in the emigration process. To achieve this, we harvested multiple organs (thymus, spleen, lymph node, liver, skin, gut, lung, and Peyer's patches) from mice over six time points of the day. We then performed quantitative fluorescence microscopy imaging assays on sections from each organ, which allowed us to minimize variability and compare expression patterns across tissues within the same mice at the same time. This approach yielded a highly tissue-specific temporal expression map for endothelial cell adhesion molecules (Figure 2A and Table S1). Integrating the profiles from all expressed molecules across all organs over time revealed a peak in expression in the evening (Figure 2B). This indicated that endothelial cells within the body (or at least within the eight organs assessed as proxy) had a distinctly higher leukocyte recruitment capacity at this time. This was in line with the negative homing data, which represented highest leukocyte emigration in the evening from blood across the whole organism (Figure 1B). Specifically, ICAM-1 was expressed in every vascular bed analyzed, VCAM-1 was expressed in all but the skin, and both exhibited peaks in expression in the evening (Figure 2C). ICAM-2 was expressed in all organs except spleen and skin, whereas P-selectin

Figure 1. Rhythmic Recruitment Is Governed by Both Microenvironment and Leukocytes

(A) Total leukocyte and leukocyte subset counts over 24 hr. Zeitgeber time (ZT, time after light onset) 1 is double plotted to facilitate viewing (n = 9–62 mice; one-way ANOVA). WBC, white blood cell; NK, natural killer; IM, inflammatory monocyte; NIM, non-inflammatory monocyte.

(B) Diagram of adoptive-transfer assay with rhythmic recipients. Shown are numbers of adoptively transferred donor cells present in the blood of recipient mice 1 hr after transfer over 24 hr. Data are normalized to ZT5 levels (n = 3–25 mice; one-way ANOVA).

(C) Diagram of adoptive-transfer assay with rhythmic donors. Shown are numbers of adoptively transferred donor cells present in blood of recipient mice 1 hr after transfer over 24 hr. Data are normalized to ZT5 levels (n = 3–17 mice; one-way ANOVA).

*$p < 0.05$, **$p < 0.01$, ***$p < 0.001$, ****$p < 0.0001$. All data are represented as mean ± SEM. See also Figure S1.

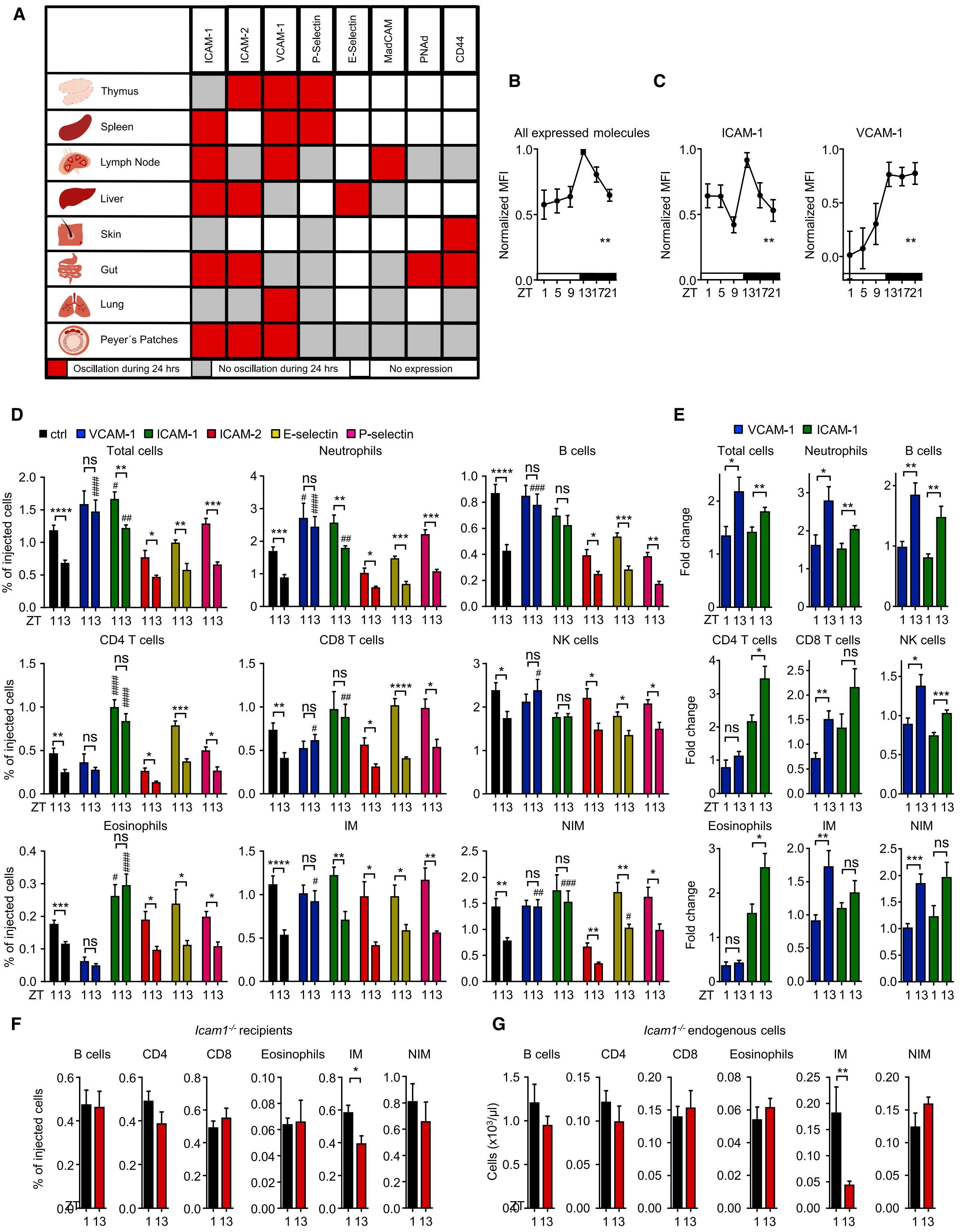

(legend on next page)

was expressed in all investigated organs but the liver. Expression for both molecules peaked in the evening; however, this was not statistically significant (Figure S2A). In functional assays, we next focused on the molecules that showed oscillations and robust expression levels across organs because these molecules were likely to be critical in mediating rhythmic homing for many of the investigated subsets. Indeed, chronopharmacological blockade with an antibody directed against VCAM-1 in the morning or at night resulted in increased numbers of adoptively transferred cells in the circulation, signifying reduced tissue homing. This additionally ablated their day-night oscillation (Figures 2D and 2E). Blockade with an anti-ICAM-1 antibody increased numbers of neutrophils, T cells, eosinophils, and non-inflammatory monocytes and ablated their rhythmicity but had no effect on inflammatory monocytes (Figures 2D and 2E). This was confirmed genetically with *Icam1*-deficient recipients (Figures 2F and 2G). Blocking of ICAM-2, E-selectin, or P-selectin, on the other hand, had little or no effect on leukocyte cellularity or oscillations in blood (Figure 2D). Importantly, we generally observed a much more pronounced blocking effect when antibodies were administered in the evening than when they were administered in the morning for both adoptively transferred and endogenous leukocyte populations (Figure 2E and Figure S2B). This established the functional importance of time of day and an oscillatory expression of endothelial cell adhesion molecules for the emigration of leukocyte subsets from blood.

Leukocyte Subset-Specific Oscillations in Pro-migratory Factors

Given that we had additionally identified rhythmicity in leukocytes to govern the emigration process (Figure 1C), we next screened blood leukocyte subsets for an oscillatory expression of adhesion molecules and chemokine receptors. Using flow-cytometry analyses across four to six time points of the day, we observed oscillations in adhesion molecules and chemokine receptors, which varied between subsets. Together, they provided a unique rhythmic molecular signature for each lineage (Figure 3A, Figures S3A–S3C, and Table S2). Focusing on the molecules that exhibited the broadest expression and most robust oscillation patterns, we performed functional blocking experiments by using antibodies or functional inhibitors. Numbers of adoptively transferred leukocytes increased in blood most prominently after injection of antibodies directed against CD49d (α4-integrin) or L-selectin (Figure 3B). In contrast, no effect was observed when PSGL-1 or the single β1- or β2-integrin subunits were blocked (Figure 3B and data not shown). Analogous to targeting endothelial cell adhesion molecules, we again observed a stronger effect when antibodies were administered in the evening than when they were administered in the morning (Figure 3B). We next assessed the functional relevance of oscillatory expression of chemokine receptors on the surface of leukocyte subtypes. Pre-treatment of morning or evening cells with pertussis toxin before adoptive transfer blocked leukocyte homing and ablated its rhythmicity, indicating leukocyte chemokine receptors to be critically involved in this process (data not shown). Specifically, strong effects on numbers and oscillations of adoptively transferred and endogenous leukocyte populations were observed when AMD3100, an antagonist against CXCR4, was administered (Figures 3C–3E and Figure S3D). In this scenario, leukocyte oscillations ceased in all assessed subtypes, which was also observed when cells were pre-treated with the antagonist *ex vivo* before adoptive transfer (Figure S3E), with the exception of inflammatory monocytes (Figure 3C). In contrast, blocking other chemokine receptors, including CXCR2 and CCR4 as well as CXCR3, CCR2, and CCR1, did not yield major effects (Figure 3C and data not shown). These data demonstrate the critical requirement of leukocyte adhesion molecules and CXCR4 in the rhythmic leukocyte migration process. In line with these findings, we observed an oscillation of *Cxcl12* mRNA expression and the CXCR4 ligand in both bone marrow and the lung (Figure S3F). Of importance, this process could be blocked pharmacologically in a time-of-day-dependent manner through the targeting of pro-migratory factors on endothelial cells or leukocytes (Figure 3F and Figure S3G).

Diurnal Homing Capacity of Leukocyte Subsets to Specific Organs

We next investigated to which organs leukocyte subsets homed over the course of the day. Adoptive transfer of morning or evening cells into phase-matched morning or evening recipients, respectively, demonstrated more leukocyte trafficking to organs in the evening, in line with our data obtained from blood (Figure 4A and Figure S4A). This excluded excessive phagocytosis or death of leukocytes at specific times as a major contributor to the diurnal effects seen in blood in the employed short time frame of 1 hr. We confirmed this by performing reciprocal homing assays where we co-injected morning or evening cells into morning or evening recipients, respectively, by using differential color labeling (Figure S4B). Specifically, we observed more homing to bone marrow, lymph node, spleen, liver, and lung (Figure 4A and Figure S4A). We observed very little homing to other investigated tissues, such as skin, thymus, and gut, in the investigated time

Figure 2. Tissue-Specific Oscillations in Endothelial Cell Adhesion Molecules

(A) Map of rhythmic protein expression of endothelial cell-adhesion molecules of various organs (n = 3–6 mice with 6 time points measured each; one-way ANOVA).

(B) Integration of all expressed molecules over all organs across the day (n = 3–6 mice with 6 time points measured each; one-way ANOVA).

(C) Integration of ICAM-1 and VCAM-1 expression over all organs across the day (n = 3–6 mice with 6 time points measured each; one-way ANOVA).

(D) Adoptive transfer of donor cells to recipients treated with functional blocking antibodies directed against the indicated molecules at ZT1 and ZT13. Results are presented as percentages of injected cells (n = 4–12 mice; one-way ANOVA followed by Dunnett comparison to control groups and unpaired Student's t test for comparisons between ZT1 and ZT13 groups).

(E) Fold change of donor cells remaining in recipient blood at ZT1 and ZT13 after anti-VCAM-1 or anti-ICAM-1 antibody treatment, respectively, in comparison with numbers of isotype antibody controls (n = 7–11 mice; unpaired Student's t test).

(F) Adoptive transfer of donor cells to $Icam1^{-/-}$ recipients at ZT1 and ZT13 (n = 6–8 mice; unpaired Student's t test).

(G) Endogenous blood leukocyte numbers in $Icam1^{-/-}$ mice at ZT1 and ZT13 (n = 6–8 mice; unpaired Student's t test).

*p < 0.05, **p < 0.01, ***p < 0.001, ****p < 0.0001; $^{\#, \#\#, \#\#\#, \#\#\#\#}$ indicate significance levels analogous to those of control groups. All data are represented as mean ± SEM. ns, not significant; MFI, mean fluorescence intensity. See also Figure S2 and Table S1.

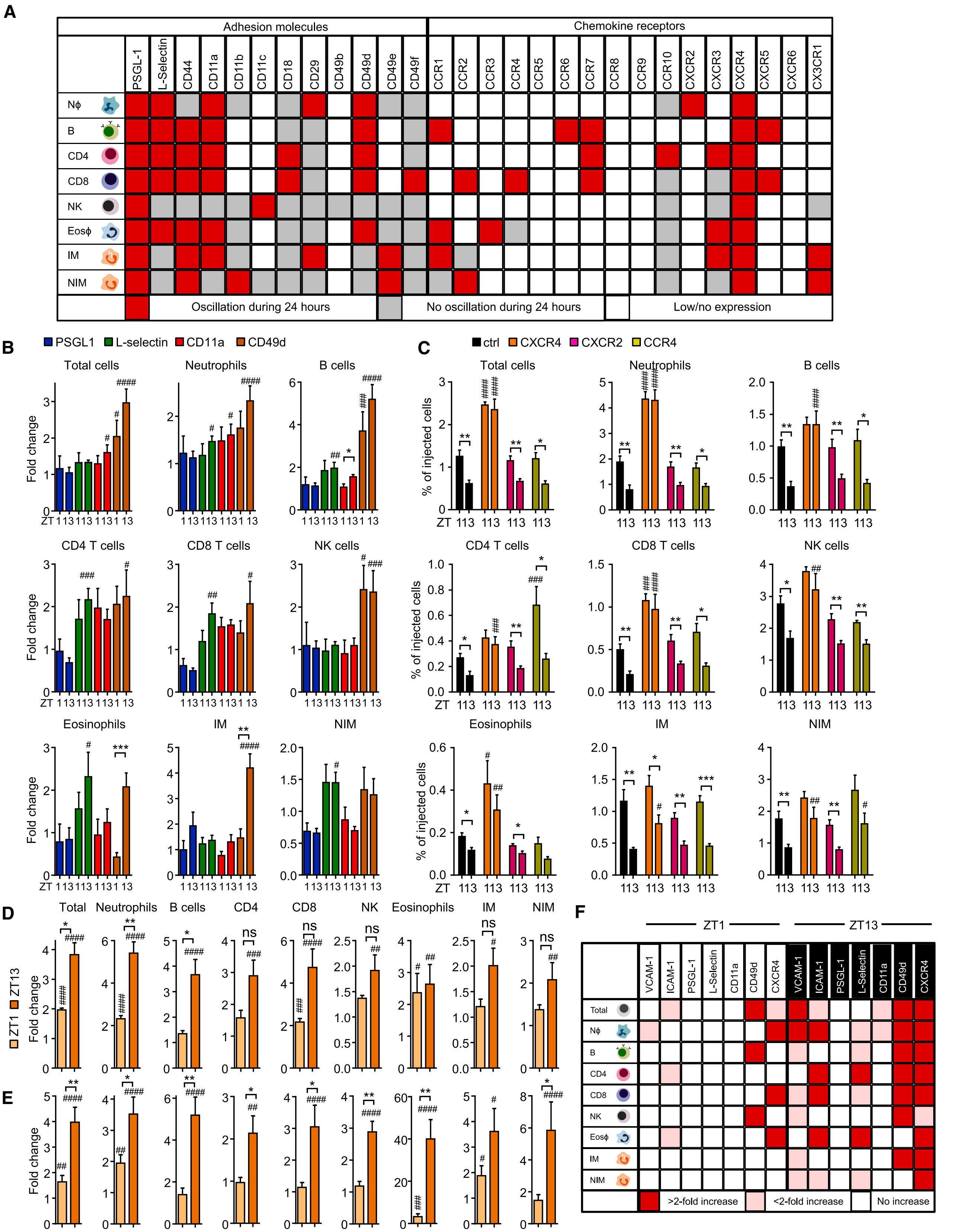

(legend on next page)

frame of 1 hr (data not shown). Each leukocyte subset exhibited a unique capacity with respect to rhythmic homing to tissues. More CD4 and CD8 T cells, B cells, and neutrophils migrated to the lymph node in the evening than in the morning (Figure S4A). To the liver, enhanced homing of inflammatory monocytes, neutrophils, B cells, and eosinophils was observed (Figure 4A). To the lung, more homing of neutrophils, inflammatory monocytes, B cells, eosinophils, and CD8 T cells was observed (Figure 4A). To the bone marrow, more homing of neutrophils, B cells, inflammatory monocytes, and NK cells was seen, (Figure S4A) and to the spleen, more homing of neutrophils, B cells, and NK cells was detected (Figure 4A).

Because homed cells could have either traversed the endothelium or remained adherent in the vasculature of the respective organ (which would both remove them from the circulation), we additionally assessed the specific location of transferred cells within tissues. To achieve this, we co-injected an anti-CD45 antibody just before perfusion and tissue harvest. This allowed us to distinguish between i.v.-CD45-labeled leukocytes (adherent cells) and non-labeled leukocytes (extravasated cells). In the liver and lung, the vast majority of transferred cells were present in the vasculature (Figure 4B). In contrast, leukocytes in bone marrow, lymph node, and spleen had predominantly traversed the endothelium (Figure 4B and Figure S4C). We confirmed these data by performing imaging analyses of organ whole mounts to visualize the precise location of cells in three dimensions (Figures 4C and 4D). This approach allowed us to additionally assess their locations with respect to organ-intrinsic structures, demonstrating that in the spleen more transferred cells were present in the red pulp than in the white pulp and that cells were extravascular in both areas (Figure 4C and Figure S4D). Together, these data clearly demonstrate a leukocyte-subset-specific capacity in the rhythmic migration to distinct organs.

Chronopharmacological Targeting of Leukocyte Homing to Tissues

We next used the identified targets among pro-migratory factors to assess which leukocyte subset was dependent on which molecule to migrate to which tissue. We built on our previous observations on the time-dependent inhibition of leukocyte emigration from blood (Figure 3F). We therefore performed the experiments in the evening (ZT13) to maximize the outcome of potential blocking effects and thus detect an influence of molecules that might not have been previously implicated in mediating the migration of leukocyte subsets to specific organs (Figures 5A–5I).

As expected for the bone marrow, anti-CXCR4 treatment had the overall strongest blocking effect on all investigated subtypes, with the exception of inflammatory monocytes (Figure 5A). Specifically, this treatment decreased numbers of extravasated cells but increased numbers of adherent leukocytes inside the vasculature, indicating a specific role in the extravasation process in this tissue (Figures 5B and 5I and Figure S5A). VCAM-1 inhibition, on the other hand, showed effects in the extravasation of CD4 and CD8 T cells and both the adhesion and extravasation of B cells given that for the latter, both extravascular and intravascular cell numbers were reduced (Figure 5A and Figure S5A). Blocking ICAM-1 reduced numbers of extravasated B cells, neutrophils, and CD8 T cells (Figure 5A).

In the lymph node, we observed the most dramatic effect with an antibody directed against L-selectin, which reduced the numbers of all investigated subsets at the step of adhesion and extravasation (Figures 5C and 5D and Figure S5B). CD11a and ICAM-1 blockade exhibited a similar, albeit slightly weaker effect, indicating their potential co-dependence in this tissue (Figures 5C and 5D and Figure S5B).

In the spleen, blockade of L-selectin exhibited the strongest effect, particularly on CD8 T cells with additional effects on B cells, neutrophils, and CD4 T cells (Figures 5E and 5F). Blocking CD11a exhibited specific effects on the ability of B cells to transmigrate given that the number of extravasated cells was reduced and the number of adherent cells was increased (Figures 5C and 5D and Figure S5C). Anti-ICAM-1 treatment inhibited B cell and inflammatory monocyte immigration (Figure 5E).

In the liver, numbers of adherent cells could be strongly reduced by interference with VCAM-1 (neutrophils, CD4 T cells, and inflammatory monocytes [IMs]), ICAM-1 (B cells, CD4 and CD8 T cells, and IMs), CXCR4 (CD4 T cells and IMs), or CD49d (IMs) (Figure 5G).

In the lung, numbers of adherent leukocytes could be reduced by blockade of VCAM-1 or ICAM-1 (all subsets except neutrophils), CXCR4, CD49d, or CD11a (B cells, CD4 T cells, and IMs) (Figure 5H).

Figure 3. Leukocyte-Subset-Specific Oscillations in Pro-migratory Molecules

(A) Map of rhythmic protein expression of adhesion molecules and chemokine receptors in blood leukocyte subsets (n = 3–6 mice with 4–6 time points measured each; one-way ANOVA).

(B) Adoptive transfer of ZT1 and ZT13 donor cells to recipients treated with functional blocking antibodies directed against the indicated molecules at ZT1 and ZT13. Cell numbers are normalized to ZT1 and ZT13 controls (n = 3–12 mice; one-way ANOVA followed by Dunnett comparison to control groups and unpaired Student's t test for comparisons between ZT1 and ZT13 groups).

(C) Adoptive transfer of donor cells to recipients treated with antagonists against the indicated molecules at ZT1 and ZT13 (n = 3–10 mice; one-way ANOVA followed by Dunnett comparison to control groups and unpaired Student's t test for comparisons between ZT1 and ZT13 groups).

(D) Fold change of donor cells remaining in recipient blood at ZT1 and ZT13 after anti-VCAM-1 and anti-ICAM-1 antibody treatment, respectively, in comparison with numbers of isotype antibody controls. (n = 3 or 4 mice; one-way ANOVA followed by Dunnett comparison to control groups and unpaired Student's t test for comparisons between ZT1 and ZT13 groups).

(E) Endogenous blood leukocyte numbers after CXCR4 antagonist treatment (n = 3 or 4 mice; one-way ANOVA followed by Dunnett comparison to control groups and unpaired Student's t test for comparisons between ZT1 and ZT13 groups).

(F) Overview of functional blocking effects on adoptively transferred leukocyte subsets in blood targeting the indicated molecules at ZT1 and ZT13 (n = 3–12 mice; one-way ANOVA followed by Dunnett comparison to control groups).

*$p < 0.05$, **$p < 0.01$, ***$p < 0.001$, ****$p < 0.0001$; $^{\#, \#\#, \#\#\#, \#\#\#\#}$ indicate significance levels analogous to those of control groups. All data are represented as mean ± SEM. ns, not significant. See also Figure S3 and Table S2.

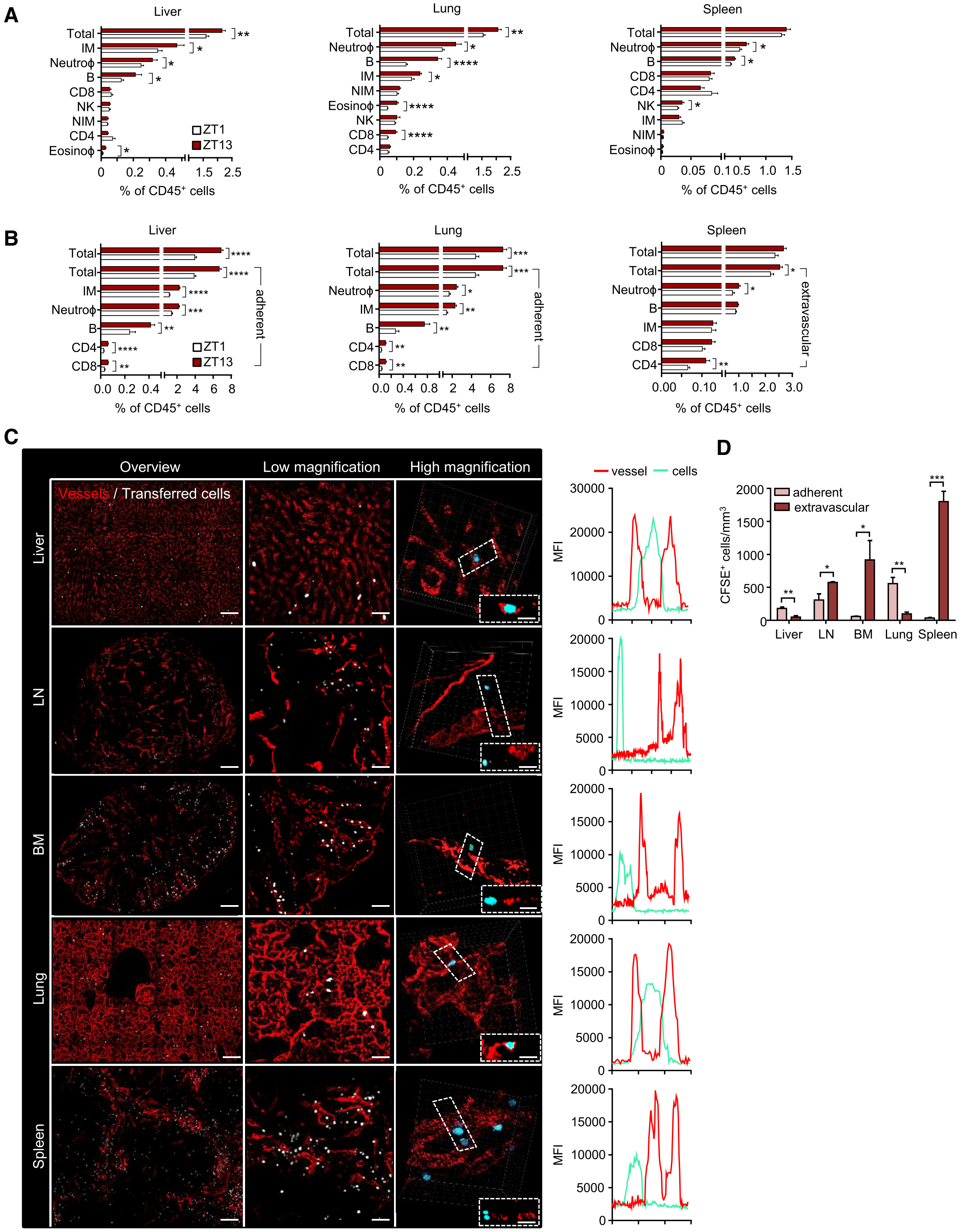

Figure 4. Diurnal Homing Capacity of Leukocyte Subsets to Specific Organs

(A) Recruitment of 10^6 adoptively transferred leukocyte subsets into liver, lung, and spleen at ZT1 and ZT13 (liver, n = 10 mice; lung, n = 10 mice; spleen, n = 24–25 mice; unpaired Student's t test).

(B) Recruitment and localization (extravascular or adherent) of 2 × 10^6 adoptively transferred leukocyte subsets into liver, lung, and spleen at ZT1 and ZT13 (n = 6–12 mice; unpaired Student's t test).

(C) Whole-mount imaging of organs defines the location of donor cells in adoptive-transfer experiments after perfusion. Boxes indicate exemplary cells whose localization within tissues is additionally shown in the z direction. Line graphs of mean fluorescence intensities (MFI) show their localization inside or outside the vasculature. Scale bars: 150 μm (overviews for liver, bone marrow [BM], lung, and spleen], 200 μm (for lymph node [LN]), 50 μm (low magnification), and 10 μm (high magnification).

(D) Quantification of numbers and localization of total transferred cells is based on whole-mount imaging of organs (n = 3 mice; unpaired Student's t test).

*$p < 0.05$, **$p < 0.01$, ***$p < 0.001$, ****$p < 0.0001$. All data are represented as mean ± SEM. See also Figure S4.

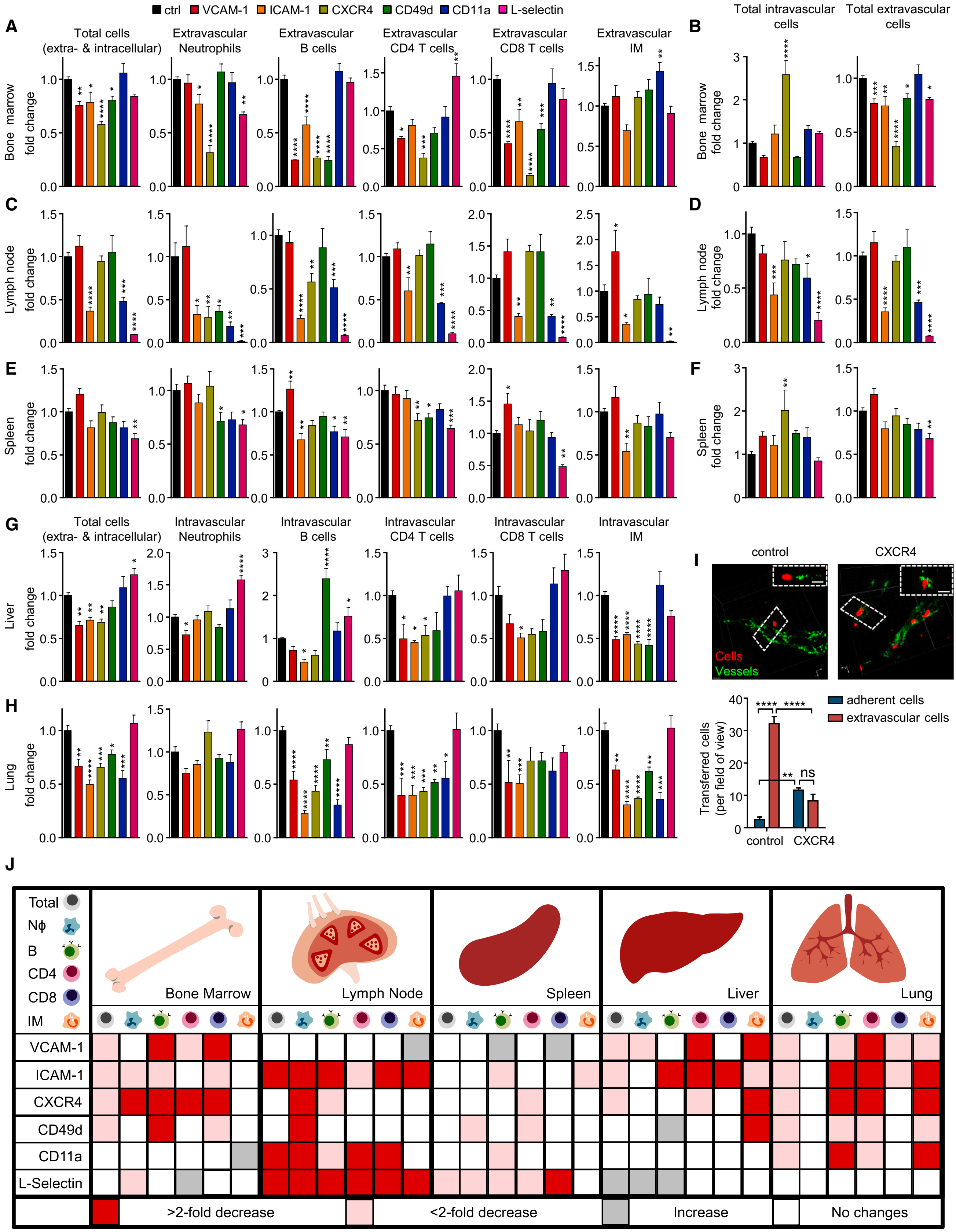

(legend on next page)

Together, these data demonstrate that pro-migratory factors on endothelial cells and leukocytes govern time-of-day-dependent migration, thereby identifying a circadian signature that determines leukocyte migration to tissues (Figure 5J and Figure S5D).

Lineage-Specific Clock Deficiency Ablates Migration Rhythms

We next investigated the relevance of a functional clock in the rhythmic trafficking behavior of immune cells. We first focused on the microenvironment by using *Cdh5*^{CreERT2}*Bmal1*$^{flox/flox}$ mice to specifically delete the circadian gene brain and muscle Arnt-like protein-1 (*Bmal1*, also known as *Arntl*) in endothelial cells (Wang et al., 2010). *Bmal1* is a core component of the cellular clockwork and the only single gene whose deficiency causes an ablation of circadian rhythmicity (Storch et al., 2007). Using these mice as recipients, we performed homing experiments and quantified the amount of adoptively transferred cells that remained in the blood. Interestingly, whereas control mice showed fewer transferred cells in the circulation in the evening, mice with *Bmal1*-deficient endothelial cells showed no difference between time points for all subsets examined (Figure 6A). We next investigated whether rhythmic homing to tissues was also ablated. Indeed, in the two organs displaying the strongest oscillations, the lung and liver, time-of-day differences were lost (Figure 6B). These observations were associated with strongly reduced evening expression of ICAM-1 and VCAM-1 in the liver and lung, respectively, of mice with *Bmal1*$^{-/-}$ endothelial cells (Figure 6C). This genetically demonstrates the relevance of oscillations in the microenvironment and indicates that within tissues, the endothelial-cell-specific clock plays a critical role in governing rhythmic leukocyte recruitment.

To assess the influence of clocks in leukocytes in this phenomenon, we used *Cd19*^{Cre}*Bmal1*$^{flox/flox}$ mice as donors to evaluate the homing capacity of clock-deficient B cells. Transferred *Bmal1*$^{-/-}$ B cells exhibited no more time-dependent homing to the spleen or lymph nodes of wild-type recipients (Figure 6D). In addition, *Bmal1*$^{-/-}$ B cells displayed diminished oscillations in the clock gene *Nr1d1* (Rev-Erbα) (Figure 6E) and reduced surface amounts of CD11a and CD49d (Figure 6F), as well as CCR7 and CXCR5 (but not CXCR4 or L-selectin) (Figure S6A and data not shown). Using *Lyz2*^{Cre}*Bmal1*$^{flox/flox}$ mice to target the clock in myeloid cells, we also observed a lack of oscillations in the migration behavior of donor neutrophils to the spleen of wild-type recipients (Figure 6G). *Bmal1*$^{-/-}$ neutrophils (Figure 6H) and monocytes (Figure S6B) displayed altered *Nr1d1* expression. Neutrophils exhibited lower expression of PSGL-1 (Figure 6I), whereas monocytes showed altered amounts of L-selectin (*Sell*), CCR2, and CD18 integrin (Figures S6B and S6C). Together, these data genetically demonstrate that both endothelial cell and leukocyte clocks are critically required for a rhythmic homing process by regulating the expression of pro-migratory factors.

Relevance of Rhythmic Leukocyte Trafficking in Inflammation and Leukemia

We next explored the relevance of oscillatory leukocyte trafficking for the immune response by using a systemic inflammatory challenge with intraperitoneally injected lipopolysaccharide (LPS). After acute stimulation, leukocyte counts in blood exhibited a dramatic drop, but time-of-day differences of leukocyte subsets, which exhibited lower numbers in the evening for all investigated subsets, were preserved (Figure 7A). This indicates the importance of rhythmic leukocyte migration for the strength of the immune response given that, indeed, tissue infiltration into the peritoneal cavity was rhythmic (Figure S7A). In addition, the administration of antibodies directed against VCAM-1, ICAM-1, or CD49d was able to block this effect in a subset-specific manner, whereas anti-CD11a treatment exhibited no effect. Even after the use of inflammatory challenge, antibodies exerted a stronger inhibitory effect on leukocyte emigration from blood at night (Figures 7A and 7B). Together, these data indicate the relevance of oscillatory leukocyte migration in determining the strength of the immune response.

We additionally explored a disease model by using a leukemia cancer model where tumor burden is measured in blood. We used both a syngeneic and a xenogeneic model of acute myeloid leukemia (AML) and B cell acute lymphoblastic leukemia (B-ALL). In the syngeneic model, CD45.1^{+} wild-type mice were injected i.v. with 5×10^6 CD45.2^{+} C1498 (AML) or BS50 (B-ALL) cells either in the morning (ZT1) or in the evening (ZT13). After 1 week, we measured numbers of circulating AML or B-ALL blasts at midday on the basis of CD45.2 expression to allow the assessment of the influence of the time of day of administration only and not the harvest. Interestingly, the rate of engraftment, defined as more than one blast per microliter, was much higher in the evening for both models (5/16 in the morning versus 13/16 in the evening), and circulating blasts were significantly higher at night than in the morning (Figure 7C). This indicates that homing and engraftment of leukemic cells is strongly time-of-day dependent. Because rhythms in the host immune response might influence tumor burden in a time-of-day-dependent manner, we additionally employed a xenogeneic model by using immune-deficient non-obese diabetic (NOD) scid *Il2rg*$^{-/-}$ (NSG) mice, which have no functional adaptive immune system and lack functional NK cells. We injected 5×10^6 NALM-6 cells, a human B-ALL cell line, either in the morning or in the evening. After 1 week, numbers of circulating human blasts at midday were significantly higher when cells had been injected at night (Figure 7D), confirming that time-of-day-dependent recruitment and engraftment are highly relevant for tumor burden.

Figure 5. Chronopharmacological Targeting of Leukocyte Homing to Tissues

(A–H) Adoptive transfer of donor cells to the bone marrow (A and B), lymph node (C and D), spleen (E and F), liver (G), and lung (H) of recipients treated with functional blocking antibodies or antagonists directed against the indicated molecules at ZT13. Cell numbers are normalized to control numbers (n = 4–8 mice; one-way ANOVA followed by Dunnett comparison to the control group). Localization of transferred cells in the bone marrow (B), lymph node (D), and spleen (F) is normalized to control localization (n = 4–8 mice; one-way ANOVA followed by Dunnett comparison to the control group).

(I) Images and quantification of localization of donor cells in bone marrow after CXCR4 blockade. Boxes indicate exemplary cells whose localization within tissues is additionally shown in the z direction (n = 3 mice; unpaired Student's t test). Scale bars: 10 μm.

(J) Overview of functional blocking effects on leukocyte recruitment to organs, targeting the indicated molecules (n = 4–8 mice; one-way ANOVA followed by Dunnett comparison to the control group).

*p < 0.05, **p < 0.01, ***p < 0.001, ****p < 0.0001. All data are represented as mean ± SEM. See also Figure S5.

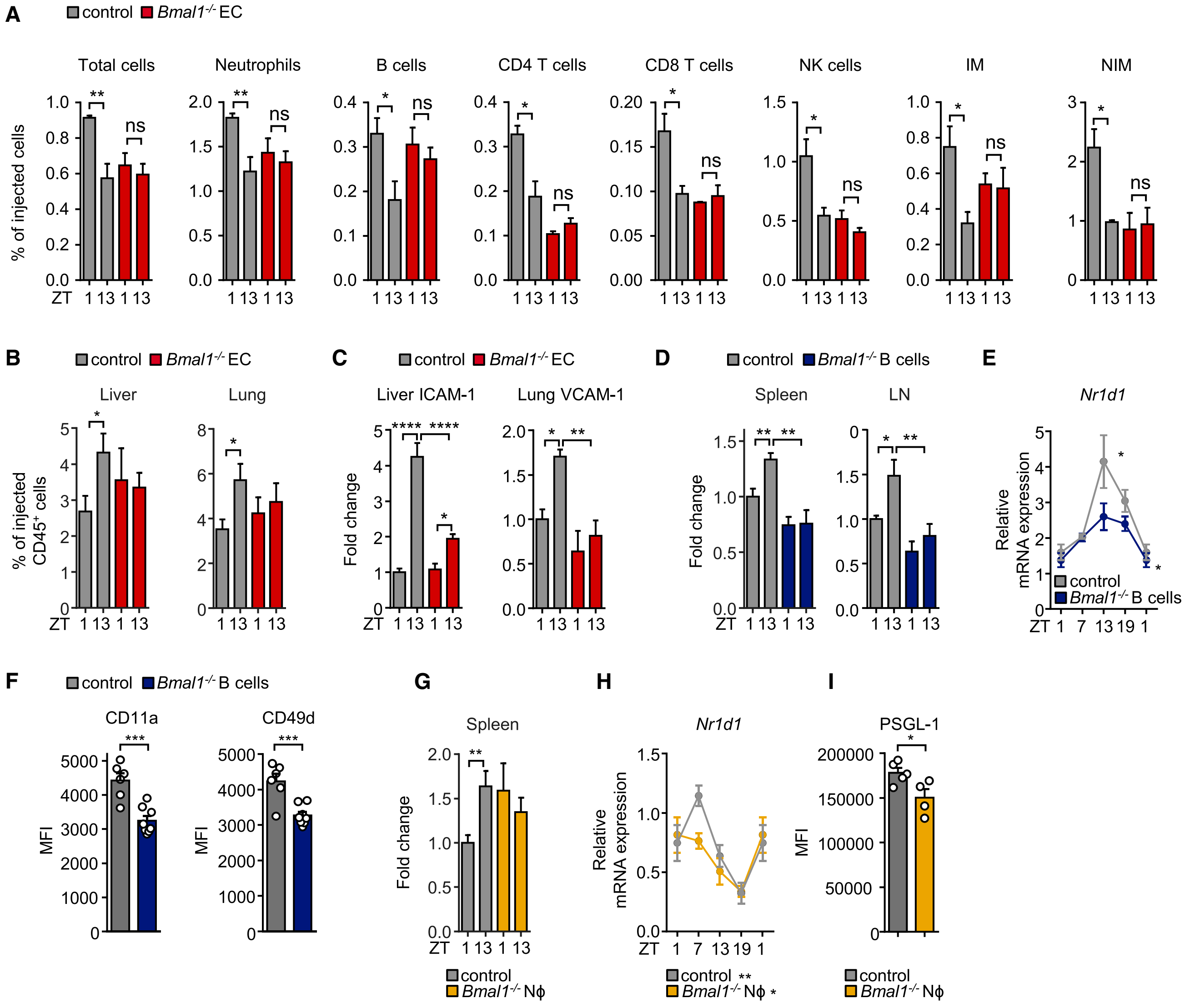

Figure 6. Lineage-Specific Clock Deficiency Ablates Migration Rhythms

(A) Numbers of adoptively transferred donor cells present in the blood of control recipients or recipients with *Bmal1*-deficient endothelial cells 1 hr after transfer at ZT1 and ZT13 (n = 3 or 4 mice; unpaired Student's t test).

(B) Adoptive transfer of donor cells to the liver and lung of control recipients or recipients with *Bmal1*-deficient endothelial cells 1 hr after transfer at ZT1 and ZT13 (n = 5 mice; unpaired Student's t test).

(C) Expression of endothelial cell ICAM-1 and VCAM-1 in liver and lung of control mice and mice with *Bmal1*-deficient endothelial cells at ZT1 and ZT13 (n = 4–7 mice; one-way ANOVA).

(D) Adoptive transfer of control or *Bmal1*-deficient B cells to the spleen and lymph node of wild-type recipients 1 hr after transfer at ZT1 and ZT13 (n = 6 mice; unpaired Student's t test).

(E) qPCR analysis of *N1rd1* mRNA expression in isolated control and *Bmal1*-deficient B cells (n = 3 mice; one-way ANOVA).

(F) CD11a and CD49d expression on control and *Bmal1*-deficient B cells in blood at ZT13 (n = 8 mice; unpaired Student's t test).

(G) Adoptive transfer of control or *Bmal1*-deficient neutrophils to the spleen of wild-type recipients 1 hr after transfer at ZT1 and ZT13 (n = 6 mice; unpaired Student's t test).

(H) qPCR analyses of *N1rd1* mRNA expression in isolated control and *Bmal1*-deficient neutrophils (n = 3 mice; one-way ANOVA).

(I) PSGL-1 expression on control and *Bmal1*-deficient neutrophils in blood at ZT13 (n = 4 or 5 mice; unpaired Student's t test).

*p < 0.05, **p < 0.01, ***p < 0.001. All data are represented as mean ± SEM. ns, not significant. See also Figure S6.

Rhythms in Human Leukocyte Migration

We finally investigated the presence of oscillations in leukocyte trafficking for humans. Using flow cytometry of human blood harvested over five time points of the day, we found that total WBC was oscillatory but had an inverted pattern compared with the one observed in mice, namely higher numbers in the evening (7 p.m.) and a trough in the morning (Figures S7B–S7C), in line with previous observations (Born et al., 1997). Within leukocyte subsets, we observed the strongest oscillations in B cells (Figure 7E) as well as CD4 and CD8 T cells (Figure S7C),

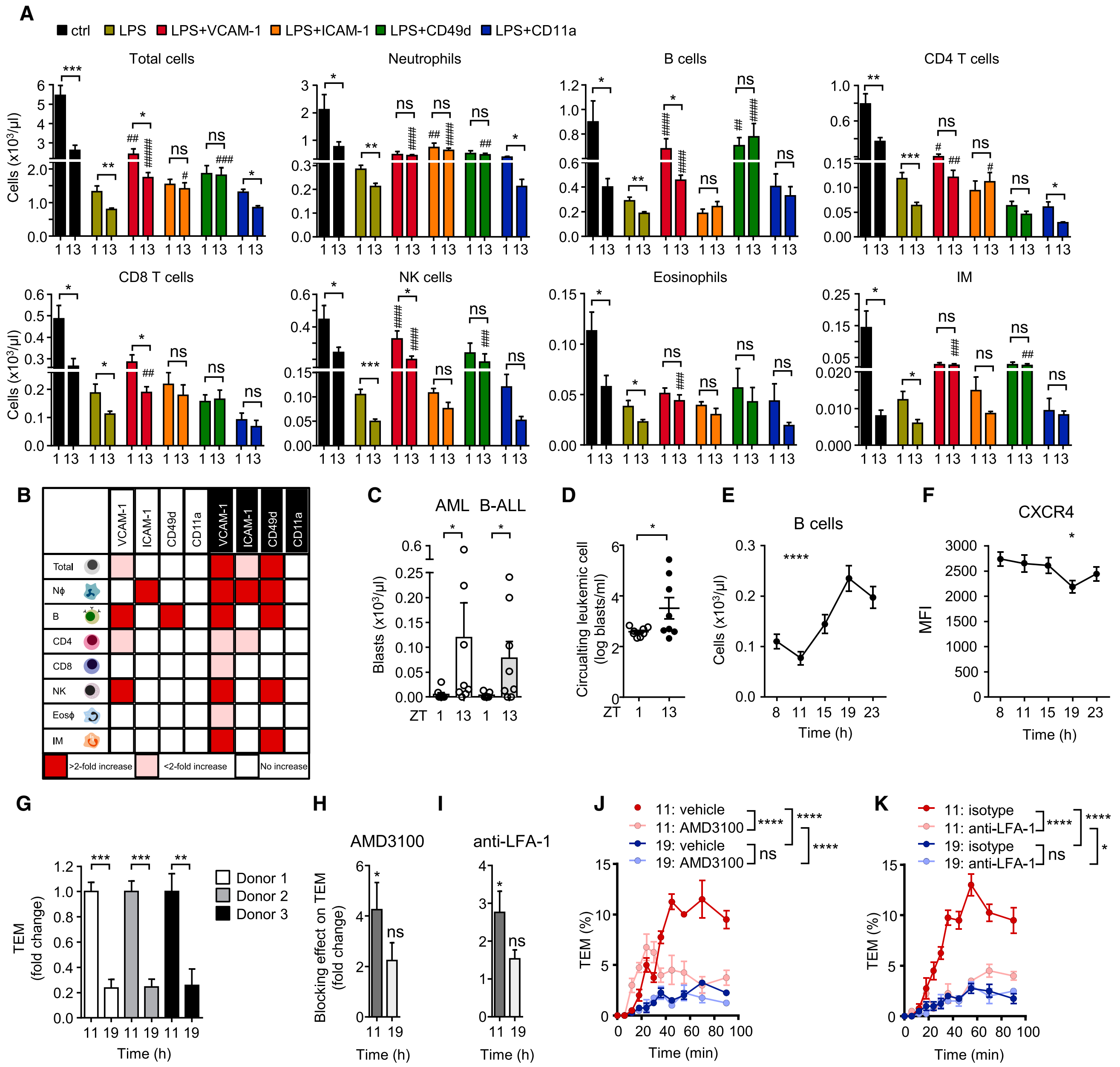

Figure 7. Relevance of Rhythmic Leukocyte Trafficking in Inflammation, Leukemia, and Humans

(A) Blood leukocyte numbers after acute treatment without (ctrl) or with LPS in combination with functional blocking antibodies directed against the indicated molecules at ZT1 and ZT13 (n = 3–12 mice; one-way ANOVA followed by Dunnett comparison to the LPS group and unpaired Student's t test for comparisons between ZT1 and ZT13 groups).

(B) Overview of functional blocking effects on leukocyte subsets in blood after LPS treatment targeting the indicated molecules at ZT1 and ZT13 (n = 3–12 mice; one-way ANOVA).

(C) Numbers of circulating blasts present in the blood of C57BL/6J CD45.1 recipients at midday 1 week after engraftment at ZT1 and ZT13 with mouse C1498 (AML) or BS50 (B-ALL) cells (n = 7 or 8 mice; Mann-Whitney test).

(D) Numbers of circulating blasts present in the blood of NSG recipient mice at midday 1 week after engraftment at ZT1 and ZT13 with human NALM-6 B-ALL cells (n = 8 mice; unpaired Student's t test).

(E) Oscillation of blood B cell numbers in human blood (n = 8 subjects; repeated-measures one-way ANOVA).

(F) CXCR4 expression on human B cells over 24 hr (n = 8 subjects; repeated-measures one-way ANOVA).

(G) Transendothelial migration (TEM) capacity of human primary B cells harvested from three donors at 11 a.m. and 7 p.m. across HUVECs. Numbers are normalized to 11 a.m. levels (n = 4 assays; unpaired Student's t test).

(H and I) Blocking efficacy of AMD3100 (H) or an anti-LFA-1 antibody (I) on TEM capacity of human primary B cells harvested at 11 a.m. and 7 p.m. Numbers are normalized to and compared with those of vehicle and isotype controls, respectively (n = 3 donors; unpaired Student's t test).

(legend continued on next page)

and other populations showed a similar trend (Figure S7C). Similar to murine cell numbers, neutrophil numbers peaked during the day (Figure S7C). With the exception of neutrophils, higher amounts of subsets in blood were inversely correlated with CXCR4 amounts (Figure 7F and Figure S7D), indicating that molecules we found to be of key importance in mice were also most likely responsible for driving rhythmic leukocyte migration processes in humans. We therefore assessed the rhythmic migratory capacity of human primary B cells (Bradfield et al., 2007) because these showed the strongest oscillations among subsets. Interestingly, blood B cells harvested in the morning exhibited a significantly higher rate of transmigration across human umbilical vein endothelial cells (HUVECs) than cells harvested from the same donors at night (Figure 7G). Strikingly, this process could be blocked efficiently in the morning by the CXCR4 antagonist AMD3100 or an antibody directed against LFA-1, whereas no significant effect was observed at night (Figures 7H–7K). Thus, these data demonstrate that human leukocytes have a rhythmic homing capacity that peaks at inverse times compared with those in mice but uses analogous molecules in the process. Altogether, we describe here a circadian signature that guides rhythmic leukocyte homing in mice and humans in steady-state, inflammation, and disease conditions.

DISCUSSION

Here, we have shown a broad and rhythmic program that governs the migration patterns of leukocyte subsets throughout the body over the course of the day. We have determined an organ- and leukocyte-subset-specific functional rhythmic signature of pro-migratory factors on endothelial cells and leukocytes. Rhythmicity in both the endothelium and leukocyte contributes to this process given that a genetically induced lack of a functional clock in either ablates time-of-day differences. We have thus identified an extensive, time-of-day-dependent trafficking zip code that guides migration of leukocytes to organs.

The process of leukocyte migration to tissues has long been studied, and multiple molecules have been implicated (Ley et al., 2007; Muller, 2016; Vestweber, 2015; Wagner and Frenette, 2008). Yet, no broad systematic approach has been undertaken for investigating the effects of multiple molecules, leukocyte subsets, and organs, particularly under non-inflammatory, steady-state conditions and with respect to the time of day. We incorporated the element of time to identify potential phases of the day when leukocyte migration to tissues and its blockade would show maximal effects. We determined this time to be the evening, or more precisely 1 hr after lights off, when mice are at the beginning of the behavioral activity phase.

The broad expression profile of VCAM-1 across all organs and its functional implications for the migration of many leukocyte subsets in steady-state conditions were unexpected given that previous studies had generally associated the molecule with inflammatory scenarios (Schnoor et al., 2015). Indeed, our data indicate that during the day, when most other studies were probably performed, VCAM-1 is hardly expressed and plays no functional role. However, expression of VCAM-1 increases over the day and exhibits a function in the evening. Thus, our approach allowed us to identify roles for molecules that had previously not been implicated in the migration of specific leukocyte subsets to organs.

For each organ and leukocyte subset, we detected a very distinct molecular homing signature. Blood leukocyte counts and bone marrow recruitment were strongly governed by CXCR4, which affected the migration of all investigated leukocyte subsets, with the exception of inflammatory monocytes. In other organs, the dependency on CXCR4 was reduced and much more subset specific. In addition to the known effect of VCAM-1 and VLA-4 in bone marrow homing, targeting ICAM-1 exhibited a broad inhibitory effect on many investigated subsets, with the exception of CD4 T cells and IMs. In addition, we also detected a role for L-selectin in neutrophil homing to this tissue. To our knowledge, ICAM-1 and L-selectin have previously not been associated with bone marrow homing given that the classical homing receptors on the endothelium consist of VCAM-1, E-selectin, and P-selectin (Mazo et al., 1998).

In the lymph node, an almost complete lack of homing was observed for all leukocyte subsets when L-selectin was blocked, in agreement with previous reports (Arbonés et al., 1994; Gallatin et al., 1983). L-selectin was also the dominant molecule regulating leukocyte migration to the spleen, particularly for CD8 T cells, which is an unexpected finding given that previous reports have not implicated a role for this molecule in this organ (Nolte et al., 2002). Also, in the lymph node we found a significant number of neutrophils and inflammatory monocytes, subsets that have generally not been investigated in this tissue under steady-state conditions (Gorlino et al., 2014; Hampton and Chtanova, 2016). Trafficking of neutrophils to the lymph node was dependent on L-selectin, CD11a, CXCR4, ICAM-1, and CD49d, whereas IMs relied mostly on L-selectin. The presence of small but detectable populations in this tissue expands the known trafficking routes of these subsets, particularly of the short-lived neutrophils. The functional relevance of their presence in steady-state conditions in lymph nodes for immune functions remains to be elucidated.

Although to our knowledge details on molecules mediating adhesion in the liver and lung under steady-state conditions are currently lacking for neutrophils and monocytes (Doyle et al., 1997; Lee and Kubes, 2008; Looney and Bhattacharya, 2014; Moreland et al., 2002; Rossaint and Zarbock, 2013), we found a small role for VCAM-1 in neutrophil recruitment in the liver. This finding is of physiological relevance because it is the likely explanation for the observed higher numbers of transferred neutrophils in blood when VCAM-1 is blocked. VCAM-1, ICAM-1, VLA-4, and CXCR4 strongly regulated homing of monocytes to

(J and K) Example of the TEM capacity of human B cells from one patient at 11 a.m. and 7 p.m. after AMD3100 (J) or anti-LFA-1 treatment (K) plotted over time (n = 4 assays; two-way ANOVA with Tukey post-test).

*p < 0.05, **p < 0.01, ***p < 0.001, ****p < 0.0001; $^{\#, \#\#, \#\#\#, \#\#\#\#}$ indicate significance levels analogous to those of the LPS groups. All data are represented as mean ± SEM. ns, not significant. See also Figure S7.

the liver, a finding very similar to that in the lung, where additional effects on LFA-1 were observed. Although the functional importance for VLA-4, VCAM-1, and ICAM-1 has been shown for CD8 T cells in the liver before (Bertolino et al., 2005; John and Crispe, 2004), we have now extended these observations to inflammatory monocytes. An interesting effect that we observed was that targeting VLA-4 increased numbers of B cells in this tissue. Also, blockade of L-selectin increased neutrophil adhesion. This indicates that in a scenario of leukocytosis induced by targeting VLA-4 (most likely due to effects in the bone marrow), B cells accumulate in the liver in an unspecific manner. In contrast, whereas blocking VCAM-1 showed a similar reduction on B cell homing to the bone marrow, more B cells accumulated in the spleen in this scenario. This demonstrates the distribution dynamics and the highly tissue- and subset-specific nature of the leukocyte homing process.

The high number of organs, leukocyte subsets, and molecules investigated prevented us from performing functional migration analyses for tissues where little homing occurred. In addition, combining the effects of multiple antibodies or antagonists to assess overlapping functions was outside the scope of this study. Our initial screening procedure in blood allowed us to detect the more dramatic, organism-wide effects, whereas smaller, tissue-specific effects might not have been detectable in some cases. We additionally focused here on events occurring at the blood-tissue interphase as the gate-keeping mechanism for leukocyte infiltration to tissues and thus did not investigate broad chemokine profiles of organs (with the exception of *Cxcl12*), something that would be strongly dependent on other tissue-resident cells, such as fibroblasts (Parsonage et al., 2005). Another important factor that we did not investigate is the heterogeneity of leukocyte subpopulations in blood. Neutrophils are probably the most heterogeneous population with respect to their age as a result of their relatively short lifespan compared with that of other subsets. Thus, most neutrophils present in blood in the morning are probably just mobilized from the bone marrow and thus represent young cells, whereas in the evening this subset has already aged significantly (Casanova-Acebes et al., 2013; Zhang et al., 2015). In contrast, most lymphocytes are a mixture of cells released from the thymus or bone marrow and cells that have reached the blood from the lymph and thus have spent a significant amount of time before in tissues. Our markers did not allow for further specification within individual leukocyte subsets. Therefore, which subsets are more prominently affected by our pharmacological interventions could not be addressed. At least for T lymphocytes, however, we have previously observed that naive, effector memory and central memory cells behave very similarly with respect to their oscillations in the blood (Druzd et al., 2017). These questions should be addressed in follow-up studies focusing on specific tissues and leukocyte subsets.

We found that time of day for antibody and antagonist administration had a great impact on their efficacy in steady-state conditions and after inflammatory challenge. In fact, for VCAM-1 and L-selectin, as well as for some subsets for CD49d, CXCR4, and ICAM-1, we hardly observed any effect in the morning. This provides a potential window for therapy with respect to targeting leukocyte trafficking at specific times. Current clinical therapeutic approaches targeting leukocyte surface molecules include natalizumab (Tysabri), a neutralizing antibody that acts against the α_4 subunit of VLA-4 and is used in the treatment of multiple sclerosis (Krumbholz et al., 2012). This drug is currently given at one time point of the day, but future studies should investigate the effect of timing drug administration in this scenario. Relapses in patients with multiple sclerosis are negatively correlated with the abundance of the night-signaling hormone melatonin in the serum (Farez et al., 2015). This is linked to a seasonal exacerbation of symptoms in the spring (Farez et al., 2015). Multiple sclerosis could additionally have a diurnal component to it given that the experimental autoimmune encephalomyelitis (EAE) animal model of multiple sclerosis shows strong time-of-day dependency in disease severity (Druzd et al., 2017; Sutton et al., 2017).

An important finding of this study is the fact that homing and engraftment capacities of leukemic cancer cells lines, both murine and human, are highly time-of-day dependent, given that administration of cells in the evening dramatically increased tumor burden a week later. This was not dependent on potential rhythmic immunogenicity of the graft because similar effects were observed in immune-deficient and immune-competent animals. Thus, time of day is an important factor for leukemia burden in mouse and human models of this disease.

These observations are of particular relevance given that we showed that rhythms in leukocyte homing extend to humans, where an inverse rhythmicity in blood leukocyte counts, particularly for lymphocyte populations, was observed. The variability between subjects was surprisingly small, given that the genetic differences between individuals are vastly greater than those between inbred mice used and that feeding and lighting schedules had not been synchronized. The strongly time-of-day-dependent transmigration capacity of human B cells could be blocked by the targeting of CXCR4, which we demonstrate to be rhythmically expressed on this subset, as well as that of LFA-1. This indicates the benefit of a chronotherapeutic approach for targeting either protein in the clinic. Indeed, targeting the CXCR4-CXCL12 axis with G-CSF has already been demonstrated to more strongly mobilize hematopoietic stem and progenitor cells in the afternoon (Lucas et al., 2008).

The inverse rhythmicity of human oscillations has thus far been linked to the altered behavioral rhythms of mice and humans such that it yields higher levels during the behavioral rest phase in both nocturnal (mice) and diurnal (humans) species. Recent data, however, demonstrate that rhythmicity in blood leukocyte counts can be decoupled from behavior and relies on reactive oxygen species in a manner independent of the microenvironment (Zhao et al., 2017). Our data demonstrate that both endothelial cells and leukocytes themselves co-govern rhythmic leukocyte migration but that lack of a clock in either is sufficient in disturbing it. The observations that a high number of pro-migratory factors display diurnal oscillations point to a role of the circadian clock in their regulation. Many of these factors exhibit binding sites for transcription factors BMAL1 and CLOCK in their promoter regions, which warrants further systematic investigations into the direct clock control of these molecules. Interplay between cell-intrinsic and -extrinsic signals appears to regulate total blood cellularity, most likely by modulating both the mobilization of cells into the circulation and the emigration, the latter of which was the subject of the present study.

STAR★METHODS

Detailed methods are provided in the online version of this paper and include the following:

- KEY RESOURCES TABLE
- CONTACT FOR REAGENT AND RESOURCE SHARING
- EXPERIMENT MODEL AND SUBJECT DETAILS
  - Mice
  - Humans
- METHOD DETAILS
  - Flow cytometry
  - Functional blocking experiments and induction of inflammation
  - Adoptive transfer assays
  - Cell isolation and Q-PCR
  - Immunofluorescence staining
  - Leukemic models
  - Transmigration assay of human B cells
- QUANTIFICATION AND STATISTICAL ANALYSIS
- DATA AND SOFTWARE AVAILABILITY

SUPPLEMENTAL INFORMATION

Supplemental Information includes seven figures and two tables and can be found with this article online at https://doi.org/10.1016/j.immuni.2018.10.007.

ACKNOWLEDGMENTS

This work was supported by the German Research Foundation (DFG) (Emmy-Noether grant SCHE 1645/2-1 and SFB914 projects B01, B09, and Z03), the German Centre for Cardiovascular Research (DZHK), and the German Ministry of Education and Research (BMBF), in addition to a European Research Council starting grant (635872, CIRCODE), the Integrated Cancer Research Site of Marseille (Inca-Inserm-DGOS 6038), and funding from the International Max Planck Research School. Additional support came from the Core Facility for Animal Models of the Ludwig Maximilians University Biomedical Center and the animal facility of the Walter Brendel Centre of Experimental Medicine. We thank Ralf Adams (Münster) and Eloi Montanez (Munich) for providing animals.

AUTHOR CONTRIBUTIONS

W.H. designed and performed experiments, analyzed results, and wrote the manuscript. S.H., S.M.H., K.K., A.d.J., J.W., D.D., C.C., L.M.I., S.B., R.P., and M.S. performed experiments. S.H. and K.K. performed imaging analyses. J.W. created the overview matrices. P.B. performed transmigration assays of human B cells. J.M.P.G., J.P., and M.A.-L. designed and performed experiments with leukemic models. C.S. designed and supervised the study, performed experiments, analyzed results, discussed data, and wrote the manuscript. All authors provided valuable inputs on the manuscript.

DECLARATION OF INTERESTS

The authors declare no competing interests.

Received: April 30, 2018
Revised: August 7, 2018
Accepted: October 2, 2018
Published: December 4, 2018

REFERENCES

Arbonés, M.L., Ord, D.C., Ley, K., Ratech, H., Maynard-Curry, C., Otten, G., Capon, D.J., and Tedder, T.F. (1994). Lymphocyte homing and leukocyte rolling and migration are impaired in L-selectin-deficient mice. Immunity *1*, 247–260.

Arjona, A., Silver, A.C., Walker, W.E., and Fikrig, E. (2012). Immunity's fourth dimension: approaching the circadian-immune connection. Trends Immunol. *33*, 607–612.

Bertolino, P., Schrage, A., Bowen, D.G., Klugewitz, K., Ghani, S., Eulenburg, K., Holz, L., Hogg, N., McCaughan, G.W., and Hamann, A. (2005). Early intrahepatic antigen-specific retention of naïve CD8+ T cells is predominantly ICAM-1/LFA-1 dependent in mice. Hepatology *42*, 1063–1071.

Born, J., Lange, T., Hansen, K., Mölle, M., and Fehm, H.L. (1997). Effects of sleep and circadian rhythm on human circulating immune cells. J. Immunol. *158*, 4454–4464.

Bradfield, P.F., Scheiermann, C., Nourshargh, S., Ody, C., Luscinskas, F.W., Rainger, G.E., Nash, G.B., Miljkovic-Licina, M., Aurrand-Lions, M., and Imhof, B.A. (2007). JAM-C regulates unidirectional monocyte transendothelial migration in inflammation. Blood *110*, 2545–2555.

Butcher, E.C. (1991). Leukocyte-endothelial cell recognition: three (or more) steps to specificity and diversity. Cell *67*, 1033–1036.

Casanova-Acebes, M., Pitaval, C., Weiss, L.A., Nombela-Arrieta, C., Chèvre, R., A-González, N., Kunisaki, Y., Zhang, D., van Rooijen, N., Silberstein, L.E., et al. (2013). Rhythmic modulation of the hematopoietic niche through neutrophil clearance. Cell *153*, 1025–1035.

Curtis, A.M., Bellet, M.M., Sassone-Corsi, P., and O'Neill, L.A. (2014). Circadian clock proteins and immunity. Immunity *40*, 178–186.

Dibner, C., Schibler, U., and Albrecht, U. (2010). The mammalian circadian timing system: organization and coordination of central and peripheral clocks. Annu. Rev. Physiol. *72*, 517–549.

Doyle, N.A., Bhagwan, S.D., Meek, B.B., Kutkoski, G.J., Steeber, D.A., Tedder, T.F., and Doerschuk, C.M. (1997). Neutrophil margination, sequestration, and emigration in the lungs of L-selectin-deficient mice. J. Clin. Invest. *99*, 526–533.

Druzd, D., Matveeva, O., Ince, L., Harrison, U., He, W., Schmal, C., Herzel, H., Tsang, A.H., Kawakami, N., Leliavski, A., et al. (2017). Lymphocyte circadian clocks control lymph node trafficking and adaptive immune responses. Immunity *46*, 120–132.

Farez, M.F., Mascanfroni, I.D., Méndez-Huergo, S.P., Yeste, A., Murugaiyan, G., Garo, L.P., Balbuena Aguirre, M.E., Patel, B., Ysrraelit, M.C., Zhu, C., et al. (2015). Melatonin contributes to the seasonality of multiple sclerosis relapses. Cell *162*, 1338–1352.

Gallatin, W.M., Weissman, I.L., and Butcher, E.C. (1983). A cell-surface molecule involved in organ-specific homing of lymphocytes. Nature *304*, 30–34.

Gorlino, C.V., Ranocchia, R.P., Harman, M.F., García, I.A., Crespo, M.I., Morón, G., Maletto, B.A., and Pistoresi-Palencia, M.C. (2014). Neutrophils exhibit differential requirements for homing molecules in their lymphatic and blood trafficking into draining lymph nodes. J. Immunol. *193*, 1966–1974.

Hampton, H.R., and Chtanova, T. (2016). The lymph node neutrophil. Semin. Immunol. *28*, 129–136.

John, B., and Crispe, I.N. (2004). Passive and active mechanisms trap activated CD8+ T cells in the liver. J. Immunol. *172*, 5222–5229.

Krumbholz, M., Derfuss, T., Hohlfeld, R., and Meinl, E. (2012). B cells and antibodies in multiple sclerosis pathogenesis and therapy. Nat. Rev. Neurol. *8*, 613–623.

Labrecque, N., and Cermakian, N. (2015). Circadian clocks in the immune system. J. Biol. Rhythms *30*, 277–290.

Lee, W.Y., and Kubes, P. (2008). Leukocyte adhesion in the liver: distinct adhesion paradigm from other organs. J. Hepatol. *48*, 504–512.

Ley, K., Laudanna, C., Cybulsky, M.I., and Nourshargh, S. (2007). Getting to the site of inflammation: the leukocyte adhesion cascade updated. Nat. Rev. Immunol. *7*, 678–689.

Looney, M.R., and Bhattacharya, J. (2014). Live imaging of the lung. Annu. Rev. Physiol. *76*, 431–445.

Lucas, D., Battista, M., Shi, P.A., Isola, L., and Frenette, P.S. (2008). Mobilized hematopoietic stem cell yield depends on species-specific circadian timing. Cell Stem Cell *3*, 364–366.

Man, K., Loudon, A., and Chawla, A. (2016). Immunity around the clock. Science *354*, 999–1003.

Marelli-Berg, F.M., Cannella, L., Dazzi, F., and Mirenda, V. (2008). The highway code of T cell trafficking. J. Pathol. *214*, 179–189.

Mazo, I.B., Gutierrez-Ramos, J.C., Frenette, P.S., Hynes, R.O., Wagner, D.D., and von Andrian, U.H. (1998). Hematopoietic progenitor cell rolling in bone marrow microvessels: parallel contributions by endothelial selectins and vascular cell adhesion molecule 1. J. Exp. Med. *188*, 465–474.

Moreland, J.G., Fuhrman, R.M., Pruessner, J.A., and Schwartz, D.A. (2002). CD11b and intercellular adhesion molecule-1 are involved in pulmonary neutrophil recruitment in lipopolysaccharide-induced airway disease. Am. J. Respir. Cell Mol. Biol. *27*, 474–480.

Muller, W.A. (2016). Transendothelial migration: unifying principles from the endothelial perspective. Immunol. Rev. *273*, 61–75.

Nguyen, K.D., Fentress, S.J., Qiu, Y., Yun, K., Cox, J.S., and Chawla, A. (2013). Circadian gene Bmal1 regulates diurnal oscillations of Ly6C(hi) inflammatory monocytes. Science *341*, 1483–1488.

Nolte, M.A., Hamann, A., Kraal, G., and Mebius, R.E. (2002). The strict regulation of lymphocyte migration to splenic white pulp does not involve common homing receptors. Immunology *106*, 299–307.

Parsonage, G., Filer, A.D., Haworth, O., Nash, G.B., Rainger, G.E., Salmon, M., and Buckley, C.D. (2005). A stromal address code defined by fibroblasts. Trends Immunol. *26*, 150–156.

Rossaint, J., and Zarbock, A. (2013). Tissue-specific neutrophil recruitment into the lung, liver, and kidney. J. Innate Immun. *5*, 348–357.

Rot, A., and von Andrian, U.H. (2004). Chemokines in innate and adaptive host defense: basic chemokinese grammar for immune cells. Annu. Rev. Immunol. *22*, 891–928.

Scheiermann, C., Kunisaki, Y., Lucas, D., Chow, A., Jang, J.E., Zhang, D., Hashimoto, D., Merad, M., and Frenette, P.S. (2012). Adrenergic nerves govern circadian leukocyte recruitment to tissues. Immunity *37*, 290–301.

Scheiermann, C., Kunisaki, Y., and Frenette, P.S. (2013). Circadian control of the immune system. Nat. Rev. Immunol. *13*, 190–198.

Scheiermann, C., Gibbs, J., Ince, L., and Loudon, A. (2018). Clocking in to immunity. Nat. Rev. Immunol. *18*, 423–437.

Schnoor, M., Alcaide, P., Voisin, M.B., and van Buul, J.D. (2015). Crossing the vascular wall: common and unique mechanisms exploited by different leukocyte subsets during extravasation. Mediators Inflamm. *2015*, 946509.

Shimba, A., Cui, G., Tani-Ichi, S., Ogawa, M., Abe, S., Okazaki, F., Kitano, S., Miyachi, H., Yamada, H., Hara, T., et al. (2018). Glucocorticoids drive diurnal oscillations in T cell distribution and responses by inducing interleukin-7 receptor and CXCR4. Immunity *48*, 286–298.e6.

Springer, T.A. (1994). Traffic signals for lymphocyte recirculation and leukocyte emigration: the multistep paradigm. Cell *76*, 301–314.

Storch, K.F., Paz, C., Signorovitch, J., Raviola, E., Pawlyk, B., Li, T., and Weitz, C.J. (2007). Intrinsic circadian clock of the mammalian retina: importance for retinal processing of visual information. Cell *130*, 730–741.

Sutton, C.E., Finlay, C.M., Raverdeau, M., Early, J.O., DeCourcey, J., Zaslona, Z., O'Neill, L.A.J., Mills, K.H.G., and Curtis, A.M. (2017). Loss of the molecular clock in myeloid cells exacerbates T cell-mediated CNS autoimmune disease. Nat. Commun. *8*, 1923.

Suzuki, K., Hayano, Y., Nakai, A., Furuta, F., and Noda, M. (2016). Adrenergic control of the adaptive immune response by diurnal lymphocyte recirculation through lymph nodes. J. Exp. Med. *213*, 2567–2574.

Vestweber, D. (2015). How leukocytes cross the vascular endothelium. Nat. Rev. Immunol. *15*, 692–704.

Wagner, D.D., and Frenette, P.S. (2008). The vessel wall and its interactions. Blood *111*, 5271–5281.

Wang, Y., Nakayama, M., Pitulescu, M.E., Schmidt, T.S., Bochenek, M.L., Sakakibara, A., Adams, S., Davy, A., Deutsch, U., Lüthi, U., et al. (2010). Ephrin-B2 controls VEGF-induced angiogenesis and lymphangiogenesis. Nature *465*, 483–486.

Zhang, D., Chen, G., Manwani, D., Mortha, A., Xu, C., Faith, J.J., Burk, R.D., Kunisaki, Y., Jang, J.E., Scheiermann, C., et al. (2015). Neutrophil ageing is regulated by the microbiome. Nature *525*, 528–532.

Zhao, Y., Liu, M., Chan, X.Y., Tan, S.Y., Subramaniam, S., Fan, Y., Loh, E., Chang, K.T.E., Tan, T.C., and Chen, Q. (2017). Uncovering the mystery of opposite circadian rhythms between mouse and human leukocytes in humanized mice. Blood *130*, 1995–2005.

STAR★METHODS

KEY RESOURCES TABLE

REAGENT or RESOURCE	SOURCE	IDENTIFIER
Antibodies		
Anti-mouse CD3, PE/DZL594, clone 17A2	Biolegend	Cat# 100246, RRID: AB_2565883
Anti-mouse CD3ε, Alexa Fluor® 488, clone 145-2C11	Biolegend	Cat# 100321, RRID: AB_389300
Anti-mouse CD4, Brilliant Violet 570, clone RM4-5	Biolegend	Cat# 100542, RRID: AB_2563051
Anti-mouse CD4, PE, clone GK1.5	Biolegend	Cat# 100408, RRID: AB_312693
Anti-mouse CD4, APC, clone GK1.5	Biolegend	Cat# 100412, RRID: AB_312697
Anti-mouse CD4, APC/Cy7, clone GK1.5	Biolegend	Cat# 100714, RRID: AB_312753
Anti-mouse CD8a, PE/Cy7, clone 53-6.7	Biolegend	Cat# 100722, RRID: AB_100722
Anti-mouse CD8a, APC/Cy7, clone 53-6.7	Biolegend	Cat# 100714, RRID: AB_312753
Anti-mouse CD8a, PE-CF594, clone 53-6.7	BD Bioscience	Cat# 562283
Anti-mouse CD8a, Alexa Fluor® 700, clone 53-6.7	Biolegend	Cat# 100730, RRID: AB_493703
Anti-mouse/human CD11b, Alexa Fluor® 700, clone M1/70	Biolegend	Cat# 101222, RRID: AB_493705
Anti-mouse CD115, PE, clone AFS98	Biolegend	Cat# 135506, RRID: AB_1937253
Anti-mouse CD115, APC, clone AFS98	eBioscience	Cat# 135510, RRID: AB_2085221
Anti-mouse Gr-1, PerCP/Cy5.5, clone RB6-8C5	Biolegend	Cat# 108428, RRID: AB_893558
Anti-mouse Gr-1, FITC, clone RB6-8C5	Biolegend	Cat# 108406, RRID: AB_313371
Anti-mouse/human CD45R/B220, Alexa Fluor® 488, clone RA3-6B2	Biolegend	Cat# 103225, RRID: AB_389308
Anti-mouse/human CD45R/B220, PE/Cy7, clone RA3-6B2	Biolegend	Cat# 103222, RRID: AB_313005
Anti-mouse/human CD45R/B220, APC-Cy7, clone RA3-6B2	Biolegend	Cat# 103224, RRID: AB_313007
Anti-mouse Siglec-F, Alexa Fluor® 647, clone E50-2440	BD Bioscience	Cat# 562680
Anti-mouse Siglec-F, APC-Cy7, clone E50-2440	BD Bioscience	Cat# 565527
Anti-mouse NK1.1, APC, clone PK136	Biolegend	Cat# 108710, RRID: AB_313397
Anti-mouse NK1.1, Alexa Fluor® 700, clone PK136	ebioscience	Cat# 56-5941-80, RRID: AB_2574504
Anti-mouse NK1.1, PE/Cy7, clone PK136	Biolegend	Cat# 108714, RRID: AB_389364
Anti-mouse NK1.1, PE/Cy5, clone PK136	Biolegend	Cat# 108716, RRID: AB_493590
Anti-mouse CD45, PE/Dazzle 594, clone 30-F11	Biolegend	Cat# 103146, RRID: AB_2564003
Anti-mouse CD45, PE, clone 30-F11	Biolegend	Cat# 103106, RRID: AB_312971
Anti-mouse CD45, APC, clone I3/2.3	Biolegend	Cat# 147708, RRID: AB_2563540
Anti-mouse CD45.1, PE-CF594, clone A20	BD Biosciences	Cat# 562452
Anti-mouse CD45.2, Alexa Fluor® 700, clone 104	Biolegend	Cat#109822, RRID: AB_493731
Anti-mouse CD45, eFluor780, clone 30-F11	eBiosciences	Cat# 47-0451-82, RRID: AB_1548781
Anti-mouse Ly6C, PE, clone HK1.4	Biolegend	Cat# 128008, RRID: AB_1186132
Anti-mouse Ly6G, PerCP/Cy5.5, clone 1A8	Biolegend	Cat# 127616, RRID: AB_1877271
Anti-mouse I-A/I-E, PE/Cy7, clone M5/114.15.2	Biolegend	Cat# 107630, RRID: AB_2069376
Anti-mouse CCR1, PE, clone 643854	R&D	Cat# FAB5986P-100
Anti-mouse CCR2, Alexa Fluor® 700, clone 475301	R&D	Cat# FAB5538N-100
Anti-mouse CCR3, FITC, clone J073E5	Biolegend	Cat# 144510, RRID: AB_2561609
Anti-mouse CCR3, PE, clone J073E5	Biolegend	Cat# 144506, RRID: AB_2561534
Anti-mouse CCR4, PE/Cy7, clone 2G12	Biolegend	Cat# 131214, RRID: AB_2244410
Anti-mouse CCR5, PE, clone HM-CCR5	Biolegend	Cat# 107006, RRID: AB_313301
Anti-mouse CCR6, PerCp/Cy5.5, clone 29-2L17	Biolegend	Cat# 129810, RRID: AB_2275515
Anti-mouse CCR7, PE, clone 4B12	Biolegend	Cat# 120106, RRID: AB_389358
Anti-mouse CCR8, PE, clone 1055c	R&D	Cat# FAB8324P-100
Anti-mouse CCR9, PE, clone 9B1	Biolegend	Cat# 129708, RRID: AB_2073249
Anti-mouse CCR10, Alexa Fluor® 700, clone 248918	R&D	Cat# FAB2815N-100

(*Continued on next page*)

Continued

REAGENT or RESOURCE	SOURCE	IDENTIFIER
Anti-mouse CXCR2, PE, clone 242216	R&D	Cat# FAB2164P-100
Anti-mouse CXCR3, PE/Cy7, clone CXCR3-173	Biolegend	Cat# 126516, RRID: AB_2245493
Anti-mouse CXCR4, PE, clone L276F12	Biolegend	Cat# 146506, RRID: AB_2562783
Anti-mouse CXCR4, PerCP/Cy5.5, clone L276F12	Biolegend	Cat# 146510, RRID: AB_2562787
Anti-mouse CXCR5, PE, clone L138D7	Biolegend	Cat# 145504, RRID: AB_2561968
Anti-mouse CXCR6, PerCP, clone 221002	R&D	Cat# FAB2145C-100
Anti-mouse CX3CR1, PerCP Cy 5.5, clone SA011F11	Biolegend	Cat# 149010, RRID: AB_2564494
Anti-mouse CD11a, PerCP/Cy5.5, clone M17/4	Biolegend	Cat# 101124, RRID: AB_2562932
Anti-mouse/human CD11b, Alexa Fluor® 700, clone M1/70	Biolegend	Cat# 101222, RRID: AB_493705
Anti-mouse CD11c, Alexa Fluor® 700, clone N418	Biolegend	Cat# 117320, RRID: AB_528736
Anti-mouse CD44, PE-CF594, clone IM7	BD Bioscience	Cat# 562464
Anti-mouse CD62L, APC/Cy7, clone MEL-14	Biolegend	Cat# 104428, RRID: AB_830799
Anti-mouse CD162, PE, clone 2PH1	BD bioscience	Cat# 555306
Anti-mouse CD18, PerCP-Cy5.5, clone C71/16	BD Bioscience	Cat# 562827
Anti-mouse/rat CD29, Alexa Fluor® 700, clone HMβ1-1	Biolegend	Cat# 102218, RRID: AB_493711
Anti-mouse CD49b, PE/Cy7, clone DX5	Biolegend	Cat# 108922, RRID: AB_2561460
Anti-mouse CD49d, PerCP/Cy5.5, clone R1-2	Biolegend	Cat# 103620, RRID: AB_2563702
Anti-mouse CD49e, PE-CF594, clone 5H10-27 (MFR5)	BD Bioscience	Cat# 564313
Anti-mouse/human CD49f, PE/Cy7, clone GoH3	Biolegend	Cat# 313622, RRID: AB_2561705
Anti-human CD8, Alexa Fluor® 488, clone HIT8a	Biolegend	Cat# 300916, RRID: AB_756152
Anti-human CD19, PE, clone HIB19	Biolegend	Cat# 302208, RRID: AB_314238
Anti-human CD56, PE/Dazzle 594, clone HCD56	Biolegend	Cat# 318348, RRID: AB_2563564
Anti-human CD14, PerCP/Cy5.5, clone HCD14	Biolegend	Cat# 325622, RRID: AB_893250
Anti-human CD16, PE/Cy7, clone 3G8	Biolegend	Cat# 302016, RRID: AB_314216
Anti-human CD49d, APC, clone 9F10	Biolegend	Cat# 304308, RRID: AB_2130041
Anti-human CD4, Alexa Fluor® 700, clone SK3	Biolegend	Cat# 344622, RRID: AB_2563150
Anti-human CXCR4, APC/Cy7, clone 12G5	Biolegend	Cat# 306528, RRID: AB_2565994
Anti-human CD3, Brilliant Violet 570, clone UCHT1	Biolegend	Cat# 300436, RRID: AB_2562124
Anti-human CD45, PE, clone HI30	BD PharMingen	Cat# 555483, RRID: AB_395875
Anti-mouse Armenian Hamster IgG, Alexa Fluor® 700, clone HTK888	Biolegend	Cat# 400926
Anti-mouse Armenian Hamster IgG, PE, clone HTK888	Biolegend	Cat# 400908
Anti-mouse Armenian Hamster IgG, Pe/Cy7, clone HTK888	Biolegend	Cat# 400922
Anti-mouse Armenian Hamster IgG, PerCp/Cy5.5, clone HTK888	Biolegend	Cat# 400932
Anti-mouse Mouse IgG 1, κ, PE, clone P3.6.2.8.1	eBioscience	Cat# 12-4714-41
Anti-mouse Rat IgG1, κ, PE, clone R3-34	BD bioscience	Cat# 553925
Anti-mouse Rat IgG2a, κ, Alexa Fluor® 488, clone RTK2758	Biolegend	Cat# 400525
Anti-mouse Rat IgG2a, κ, Alexa Fluor® 700, clone eBR2a	eBioscience	Cat# 56-4321-80
Anti-mouse Rat IgG2a, k, APC/Cy7, clone RTK2758	Biolegend	Cat# 400524
Anti-mouse Rat IgG2a, κ, PE, clone RTK2758	Biolegend	Cat# 400508
Anti-mouse Rat IgG2a, κ, PE-CF594, clone R35-95	BD Bioscience	Cat# 562302
Anti-mouse Rat IgG2a, κ, PE/Cy7, clone RTK2758	Biolegend	Cat# 400522
Anti-mouse Rat IgG2a, κ, PerCp/Cy5.5, clone RTK2758	Biolegend	Cat# 400532
Anti-mouse Mouse IgG2a, κ, PerCp/Cy5.5, clone MOPC-173	Biolegend	Cat# 400258
Anti-mouse Rat IgG2b, κ, Alexa Fluor® 700, clone RTK4530	Biolegend	Cat# 400628
Anti-mouse Rat IgG2b, κ, PE, clone RTK4530	Biolegend	Cat# 400610
Anti-mouse Rat IgG2b, κ, PE/Cy7, clone RTK4530	Biolegend	Cat# 400618
Anti-mouse Rat IgG2b, κ, PE CP594, clone A95-1	BD Bioscience	Cat# 562308
Anti-mouse Rat IgG2b, κ, PerCP/Cy5.5, clone RTK4530	Biolegend	Cat# 400632
Anti-mouse ICAM-1, PE, clone YN1/1.7.4	Biolegend	Cat# 116108, RRID: AB_313699

(*Continued on next page*)

Continued

REAGENT or RESOURCE	SOURCE	IDENTIFIER
Anti-mouse VCAM-1, PE, clone 429 (MVCAM.A)	Biolegend	Cat# 105714, RRID: AB_1134164
Anti-mouse E-selectin, PE, clone 10E9.6 (RUO)	BD Bioscience	Cat# 553751
Anti-mouse/human P-selectin, PE, clone Psel.KO2.3	eBioscience	Cat# 2-0626-80, RRID: AB_1210864
Anti-mouse MadCAM, Alexa Fluor® 488, clone MECA-367	Biolegend	Cat# 120708, RRID: AB_493398
Anti-mouse ICAM-2, Alexa Fluor® 488, clone 3C4 (MIC2/4)	Biolegend	Cat# 105609, RRID: AB_2264501
Anti-mouse/human PNAd, Biotin, clone MECA-79	Biolegend	Cat# 120804, RRID: AB_493557
Anti-mouse PECAM-1, Alexa Fluor® 647, clone MEC13.3	Biolegend	Cat# 102516, RRID: AB_2161029
Anti-mouse PECAM-1, APC, clone 390	Biolegend	Cat# 102410, RRID: AB_312905
Anti-mouse/human CD44, PE, clone IM7	Biolegend	Cat# 103008, RRID: AB_312959
Streptavidin, Cy3	Biolegend	Cat# 405215
Anti-mouse Rat IgM, κ, Biotin, clone RTK2118	Biolegend	Cat# 400804
Anti-mouse Rat IgG2a, κ, PE, clone RTK2758	Biolegend	Cat# 400508
Anti-mouse Rat IgG2b, κ, PE, clone RTK4530	Biolegend	Cat# 400608
Anti-mouse Rat IgG2a, κ, Alexa Fluor® 488, clone RTK2758	Biolegend	Cat# 400525
Anti-mouse Rat IgG1, κ, PE, clone P3.6.2.8.1	eBioscience	Cat# 12-4714-82
Anti-mouse anti-ICAM-1, clone YN1/1.7.4	BioXcell	Cat# BE0020-1, RRID: AB_1107661
Anti-mouse anti-ICAM-2, clone 3C4(mIC2/4)	BD bioscience	Cat# 553325
Anti-mouse anti-VCAM-1, clone M/K-2.7	BioXcell	Cat# BE0027, RRID: AB_1107572
Anti-mouse anti-P-selectin, clone RB40.34	BD bioscience	Cat# 553742
Anti-mouse anti-CD62E, clone 10E9.6	BD bioscience	Cat# 553749
Anti-mouse anti-CD62L, clone Mel-14	BioXcell	Cat# BE0021, RRID: AB_1107665
Anti-mouse anti-PSGL-1, clone 4RA10	BioXcell	Cat# BE0186, RRID: AB_10950305
Anti-mouse anti-CD18, clone M18/2	BioXcell	Cat# BE0009, RRID: AB_1107607
Anti-mouse anti-CD29, clone KMI6	BioXcell	Cat# BE0232, RRID: AB_2687714
Anti-mouse anti-CD49d, clone clone PS/2	BioXcell	Cat# BE0071, RRID: AB_1107657
Anti-mouse anti-CD11a, clone Clone: M17/4	BioXcell	Cat# BE0006, RRID: AB_1107578
Anti-mouse Rat IgG2a Isotype control, clone 2A3	BioXcell	Cat# BE0089, RRID: AB_1107769
Anti-mouse Rat IgG1 Isotype control, clone HRPN	BioXcell	Cat# BE0088, RRID: AB_1107775
Anti-mouse Rat IgG2b Isotype control, clone LTF-2	BioXcell	Cat# BE0090, RRID: AB_1107780
Chemicals, Peptides, and Recombinant Proteins		
CCR4 antagonist, C 021 dihydrochloride	Tocris	Cat# 3581
CXCR2 antagonist, SB 265610	Tocris	Cat# 2724
CXCR4 antagonist, AMD 3100 octahydrochloride	Tocris	Cat# 3299
CellTrace CFSE	Thermo Fisher Scientific	Cat# C34554
Cell tracker Deep red	Thermo Fisher Scientific	Cat# C34565
Lipopolysaccharides (LPS)	Sigma	Cat# L4516
Tween80	Sigma	Cat# P4780
Dimethyl sulfoxide (DMSO)	Sigma	Cat# D2650
Collagenase IV	Sigma	Cat# C5138
Deoxyribonuclease I (Dnase I)	Aplicem	Cat# A3778
DAPI	Biolegend	Cat# 422801
Tamoxifen	Sigma	Cat# T5648
TNFα	Peprotech	Cat# 300-01A
IFNγ	Peprotech	Cat# 300-02
CXCL12	MerckSerono	In-house production
Critical Commercial Assays		
EasySep mouse neutrophil enrichment Kit	STEMCELL Technologies	Cat# 19762
EasySep mouse monocyte isolation Kit	STEMCELL Technologies	Cat# 19861
EasySep mouse B cell isolation kit	STEMCELL Technologies	Cat# 19854

(*Continued on next page*)

Continued

REAGENT or RESOURCE	SOURCE	IDENTIFIER
RNeasy Plus mini Kit	QIAGEN Hilden Germany	Cat# 74136
Human B cell negative selection kit	Miltenyi Biotec	Cat# 130-091-151
Experimental Models: Cell Lines		
BS50 B-ALL cells	Rudi W Hendriks, Erasmus MC Rotterdam, the Netherlands	N/A
C1498 AML cells	ATCC	Cat# TIB49
NALM-6 human B-ALL cells	ATCC	Cat# CRL-3273
human umbilical vein endothelial cells	University Geneva Hospital	N/A
Experimental Models: Organisms/Strains		
CD19-cre mice	Jackson Laboratories	Cat# 006785
Lyz2-cre mice	Jackson Laboratories	Cat# 004781
Bmal1$^{flox/flox}$	Jackson Laboratories	Cat# 007668
Cdh5-cre/ERT2 mice	Ralf Adams, MPI Münster, Germany	N/A
C57BL/6J CD45.1 (B6.SJL-*Ptprc*^{a}*Pepc*^{b}/BoyCrl)	Charles River	Ly5.1 mice
NSG (NOD.Cg-*Prkdc*^{scid}*IL2rg*^{tm1Wjl}/SzJ)	Charles River	JAX Cat# 05557
Oligonucleotides		
Nr1d1 Forward GAT AGC TCC CCT TCT TCT GCA TCA TC	Eurofins Genomics	N/A
Nr1d1 Reverse TTC CAT GGC CAC TTG TAG ACT TC	Eurofins Genomics	N/A
Sell Forward GAC GCC TGT CAC AAA CGA AA	Eurofins Genomics	N/A
Sell Reverse GCC CGT AAT ACC CTG CAT CA	Eurofins Genomics	N/A
Cxcl12 Forward CAG AGC CAA CGT CAA GCA	Eurofins Genomics	N/A
Cxcl12 Reverse AGG TAC TCT TGG ATC CAC	Eurofins Genomics	N/A
Software and Algorithms		
GraphPad Prism7	Graphpad software	N/A
Flowjo 10.4	Flowjo, LLC	www.flowjo.com
Becton Dickinson	FACSDiva v8.0.1	N/A
Zeiss	ZEN	N/A
ImageJ version 1.51n	https://imagej.net	N/A
slidebook version 6	Intelligent Imaging Innovations, 3i	www.intelligent-imaging.com

CONTACT FOR REAGENT AND RESOURCE SHARING

Reagents used in this study are available from the commercial sources listed. Further information and requests for other materials should be directed to and will be fulfilled by the Lead Contact, Christoph Scheiermann (christoph.scheiermann@med.uni-muenchen.de or christoph.scheiermann@unige.ch)

EXPERIMENT MODEL AND SUBJECT DETAILS

Mice

Male C57BL/6N mice aged 7–8 weeks were purchased from Charles River Laboratories (Sulzfeld, Germany). *Bmal1*$^{flox/flox}$, *Cd19cre*, *Lyz2cre* transgenic mice were purchased from Jackson Laboratories, and crossbred to target B cells and myeloid cells, respectively. *Cdh5-creERT2* mice were obtained as a gift from Ralf Adams (Max-Planck-Institute for Molecular Biomedicine, Münster) via Eloi Montanez (LMU, Munich) and were given intraperitoneal tamoxifen injections for five consecutive days to induce *Cre* recombinase expression. Mice were then used for experiments 2–3 weeks after. NSG and C57BL/6J CD45.1 mice were obtained from Jackson Laboratory and Charles River respectively and bred in a pathogen-free environment. Experimental mice were male and used at 6–12 weeks of age. Mice were maintained in a 12 h light: 12 h dark cycle with *ad libitum* access to food and water. For some experiments, mice were put in cabinets to change the light phase in order to perform experiments with animals on different light schedules at the same time. All animal procedures were in accordance with the German Law of Animal Welfare or the French laws and protocols and approved by the Regierung of Oberbayern or French animal ethics committees, respectively.

Humans

Eight healthy volunteers (four males and four females) aged 25-40 years donated blood for human blood counts experiment. Three healthy volunteers aged 26–48 years donated blood for the human B cell transmigration assay. Experiments were approved by the ethics committee of the LMU Munich and the University of Geneva. All volunteers gave written consent to participating in the study.

METHOD DETAILS

Flow cytometry

Mice were anesthetized by inhalation of isoflurane. Blood was collected by bleeding into EDTA-coated capillary tubes. Leukocyte counts were obtained using an IDEXX ProCyte DX cell counter. Erythrocytes were lysed by red blood cell (RBC) lysis buffer (0.8% NH_4Cl) 2 times, for 5 min each. Abdominal fluid was collected with a syringe by flushing with 5 mL PBS. Spleens were harvested from animals and processed through a cell strainer (40 μm, Thermo Fisher Scientific). Bone marrow cells were harvested from either one femur only or two femurs and two tibias by flushing the bone gently with cold PBS. Lung and liver were first cut into small pieces in DPBS, supplemented with calcium and magnesium (Sigma) and then incubated for 1 h in digestion buffer with collagenase IV (1 mg/ml, C5138, Sigma) and DNase I (0.2 mg/ml, Applichem) at 37°C with gentle agitation. After digestion, cells were filtered through a cell strainer (40 μm, Thermo Fisher Scientific) and resuspended in 5 mL RBC lysis buffer for 5 min. After centrifugation, the supernatant was removed. Leukocytes were resuspended in PBS supplemented with 2% fetal bovine serum (GIBCO) and 2mM EDTA, then stained with fluorescence-conjugated antibodies for 30 min on ice. After washing with PBS, cells were resuspended in DAPI (Biolegend) buffer, and analyzed by flow cytometry using a Gallios Flow Cytometer (Beckman Coulter).

Human blood was collected into EDTA-coated tubes (SARSTEDT, Germany) at 8am, 11am, 3pm, 7pm and 11pm. Human blood was prepared as described above for mouse cells and stained with antibodies at room temperature for 30 min. After washing with PBS, cells were resuspended in DAPI (Biolegend) buffer, and analyzed by flow cytometry using a Gallios Flow Cytometer (Beckman Coulter).

Functional blocking experiments and induction of inflammation

To investigate the role of specific pro-migratory molecules in the rhythmic homing process, antibody or antagonist experiments were performed in combination with adoptive transfer assays. Blocking antibodies or chemokine antagonists were diluted into working concentrations (see table below) with PBS or 5% DMSO with 1% Tween80 (Sigma) and injected i.v. or i.p. to recipient mice 2 h before injection of donor cells. Cells were then processed as described above. In order to induce systemic inflammatory conditions, LPS (L4516, Sigma) was injected i.p. (10 mg/kg) to recipient mice at the same time as the injection of blocking antibodies. For CXCR4 *ex vivo* blocking, donor cells were pre-incubated with AMD3100 (300ug/ml) for 1 hour at 37°C in RPMI 1640 (Sigma) plus 10% FCS (Sigma) and stained with CFSE for 20 min.

Working concentration of blocking antibodies and functional blockers.

Antibodies or chemicals	Volume	medium	Injection
anti-ICAM-1	200 μg/mouse	PBS	i.v.
anti-ICAM-2	60 μg/mouse	PBS	i.v.
anti-VCAM-1	200 μg/mouse	PBS	i.v.
anti-P-selectin	30 μg/mouse	PBS	i.v.
anti-CD62E	50 μg/mouse	PBS	i.v.
anti-CD62L	200 μg/mouse	PBS	i.v.
anti-PSGL-1	200 μg/mouse	PBS	i.v.
anti-CD18	200 μg/mouse	PBS	i.v.
anti-CD29	200 μg/mouse	PBS	i.v.
anti-CD49d	100 μg/mouse	PBS	i.v.
anti-CD11a	100 μg/mouse	PBS	i.v.
CCR4 antagonist	125 μg/mouse	PBS	i.p.
CXCR2 antagonist	125 μg/mouse	5% DMSO with 1% Tween80	i.p.
CXCR4 antagonist	125 μg/mouse	PBS	i.p.

Adoptive transfer assays

To investigate the emigration of leukocytes from blood, adoptive transfer experiments were performed and donor cells remaining in blood were measured as a negative indicator of how many cells had migrated into tissues. First, donor cells were obtained from bone marrow and spleen from donor mice. Single cell suspensions were obtained by flushing bone marrow with cold PBS and smashing spleen gently through a cell strainer (40 μm, Thermo Fisher Scientific). Cells were lysed with RBC lysis buffer for 5 min and resuspended in cell incubation buffer (PBS, 0.2%BSA, 2mM EDTA) and counted on a cell counter (ProCyte DX cell counter).

10^7 bone marrow cells and 10^7 spleen cells were mixed as donor cells for one recipient mouse. Donor cells were labeled with 1.5 μM CFSE (Thermo Fisher Scientific) or 0.1 μM CellTracker Deep Red dye (Thermo Fisher Scientific) for 20 min at 37°C. In adoptive transfer experiments using donors of different phases, donor cells from two time zones (10^7 mixed donor cells per time zone) were labeled differently and injected into one recipient. After one hour, blood and organs were harvested from recipient mice and processed as described above for flow cytometry analyses.

In some experiments, injections of an anti-CD45 antibody (clone I3/2.3) were followed by perfusion in order to distinguish between cells adherent to the vascular endothelium or located in the extravascular space. Injection of donor cells was performed as described before. After 56 min, 10 μl anti-CD45 (clone I3/2.3) in 200 μl PBS were injected intravenously to recipient mice. 4 min later, mice were sacrificed using an overdose of isoflurane and perfused with PBS via first the left ventricle and then the right ventricle in order to perfuse the whole body and the lung, respectively.

Cell isolation and Q-PCR

Spleen B cells were purified from *Cd19*cre *Bmal1*$^{flox/flox}$ and littermate control mice using the EasySep mouse B cell isolation kit (STEMCELL Technologies) according to the manufacturer's protocol, and purity (> 92%) was accessed by flow cytometry. Bone marrow monocytes and neutrophils were purified from *Lyz2*cre *Bmal1*$^{flox/flox}$ and littermate control mice using a monocyte isolation kit and a neutrophil enrichment kit (STEMCELL Technologies), respectively. Purity of monocytes was about > 93% and of neutrophils around > 82%.

RNA was extracted from isolated cells using the RNeasy Plus mini Kit (QIAGEN, Hilden Germany) following the manufacturers' instructions. Total organ RNA extraction was performed with TRIzol (QIAGEN). Tissues were homogenized with a homogenizer (SpeedMill PLUS, Analytic Jena). RNA clean-up was performed using the RNeasy Plus mini Kit (QIAGEN, Hilden Germany) and following the manufacturers' instructions. RNA samples were analyzed using a NanoDrop2000 (Thermo Scientific) to determine RNA concentration and quality. RNA was stored at −80°C. For reverse transcription, 150-200 ng RNA for isolated cells and 2 μg RNA for organs were used with the High Capacity cDNA Reverse Transcription Kit (Applied Biosystems). cDNA samples were stored at −20°C prior to use in quantitative PCR (Q-PCR). Q-PCR was performed with a StepOnePlus Real-Time PCR System (Applied Biosystems) in 96-well plates with SYBR green compatible primers at 60°C. Duplicates or triplicates were performed for each Q-PCR sample. The total reaction volume was 10 μl, containing 5 μl SYBR green, 1 μl primer mix (5 μM), 2 μl H_2O and 1.5 ng cDNA for cells or 20ng cDNA for organs. Gene expression levels were normalized to the housekeeping gene *Gapdh*.

Immunofluorescence staining

To measure adhesion molecule expression levels on endothelial cells, organs were placed in OCT (TissueTec), frozen at −80°C and sectioned with a thickness of 10 μm on a cryostat (Leica). Sections were fixed with cold methanol for 10 min at room temperature, incubated in PBS containing Triton X-100 (0.5%), and normal goat serum (20%). Sections were stained with antibodies and incubated at 4°C overnight. Images were obtained using a Zeiss Axio Examiner.D1 microscope equipped with 405, 488, 563, and 655 nm LED excitation light sources. All quantifications were performed using mask analyses with the Zeiss software based on PECAM-1 expression. Quantifying expression of other fluorescent channels within this mask was then performed. Areas smaller than 10 μm^2 were excluded from analysis to minimize non-specific signals. Protein expression levels were presented as mean fluorescence intensity (MFI) within the mask area and after subtraction of the respective isotype controls. Levels of expression below the isotype threshold for all assessed time-points or the majority of time-points was termed no or low expression, respectively.

For visualizing the precise localization of injected donor cells in adoptive transfer assays, mice were injected intravenously with donor cells and additionally with 40 μl of an anti-PECAM-1 antibody (clone 390) in 160 μl PBS prior to organ harvest. 5 min later, mice were anesthetized by inhalation of isoflurane, and perfused with PBS. Organs were put in OCT and frozen at −80°C. Images were obtained using a Zeiss Axio Examiner.Z1 confocal spinning disk microscope equipped with 405, 488, 561, and 640 nm laser sources using both tissue sections and whole mounts of organs. Quantification was performed using ImageJ (version 1.51n) and slidebook (version 6, Intelligent Imaging Innovations, 3i).

Leukemic models

To assess leukemic engraftment, adult NSG or C57BL/6J CD45.1 mice (6–10 weeks old) were injected retro-orbitally with 5 x10^6 leukemic cells at ZT1 or ZT13. Leukemic development was monitored on blood samples obtained by bleeding into Heparin-coated capillary tubes. Leukocyte counts were obtained using an IDEXX ProCyte DX cell counter and percentage of leukemic cells was determined by flow cytometry. Samples from mice engrafted with BS50 and C1498 leukemic cells were stained with anti-mouse CD45.1-PE-CF594 (clone A20, BD Biosciences) and anti-mouse CD45.2-AF700 (clone 104, Biolegend), while samples from NSG mice engrafted with NALM6 were stained with anti-human CD45-PE (BD PharMingen) and anti-mouse CD45-eFluor780 (clone 30-F11, eBiosciences). RBC were lysed using BD FACs Lysis buffer and samples were analyzed using a Fortessa (Becton Dickinson) flow cytometer.

Transmigration assay of human B cells

Non-synchronized HUVECs were cultured in chamber slides for 2–3 days and then treated for 24 h using a chronic activation protocol (Bradfield et al., 2007). The first stage of activation consisted of overnight TNFα (1000 U/ml) and IFNγ (500 U/ml) stimulation. B cells (80%–95%) were purified from EDTA-treated blood collected from healthy donors using a negative selection kit (Miltenyi Biotec).

The flow assay set-up consisted of a heated microscope chamber (37°C) and a calibrated pump where flow was generated over attached HUVEC monolayers by perfusing wash buffer, or a B cell suspension. The flow rate was set to represent small venules/capillaries (0.05 Pa). Assays were initiated with a second stage HUVEC activation, where CXCL12 (1 μM) was perfused over the monolayer for 15 min (step 1). Wash-buffer was then pumped for 10 min over the HUVECs to remove any unbound CXCL12 before the B cell suspension was perfused over the HUVECs for 5 min (step 2) followed by 90 min of wash-buffer (step 3). Throughout steps 2–3, images of the captured B cells were taken using phase-contrast microscopy, and a high-resolution camera. Individual images were recorded every 30 s and compiled into short movie sequences, allowing analysis of individual B cells over large areas. B cells adherent to the surface of the HUVECs showed a phase-white appearance, whereas those that had transmigrated showed a phase-black appearance. Adhesion events were recorded as the total of number of cells per unit field (mm^2). Transmigration events were presented as a percentage of total B cells captured from flow per unit field. All experiments were carried out using quadruplicate fields and presented as a mean value with + standard error measurements (±SEM).

QUANTIFICATION AND STATISTICAL ANALYSIS

Data was analyzed using Prism 7 (GraphPad) and presented as mean ± standard error of mean (SEM). A p value < 0.05 was considered as statistically significant. Comparisons between two groups were performed using unpaired Student's t test. One-way ANOVA analysis followed by Tukey's multiple comparison test was used for multiple group comparison. One-way ANOVA analysis followed by Dunnett's test was used for comparison between control and treatment groups. Human WBC counts were analyzed by repeated-measures one-way ANOVA. Mann-Whitney non-parametric analyses were performed for non-Gaussian distribution patterns in leukemia tumor burden.

DATA AND SOFTWARE AVAILABILITY

Data are available upon request.

Immunity, Volume *49*

Supplemental Information

Circadian Expression of Migratory Factors Establishes Lineage-Specific Signatures that Guide the Homing of Leukocyte Subsets to Tissues

Wenyan He, Stephan Holtkamp, Sophia Martina Hergenhan, Kerstin Kraus, Alba de Juan, Jasmin Weber, Paul Bradfield, Julien Martin Pierre Grenier, Jeoffrey Pelletier, David Druzd, Chien-Sin Chen, Louise Madeleine Ince, Susanne Bierschenk, Robert Pick, Markus Sperandio, Michel Aurrand-Lions, and Christoph Scheiermann

Figure S1

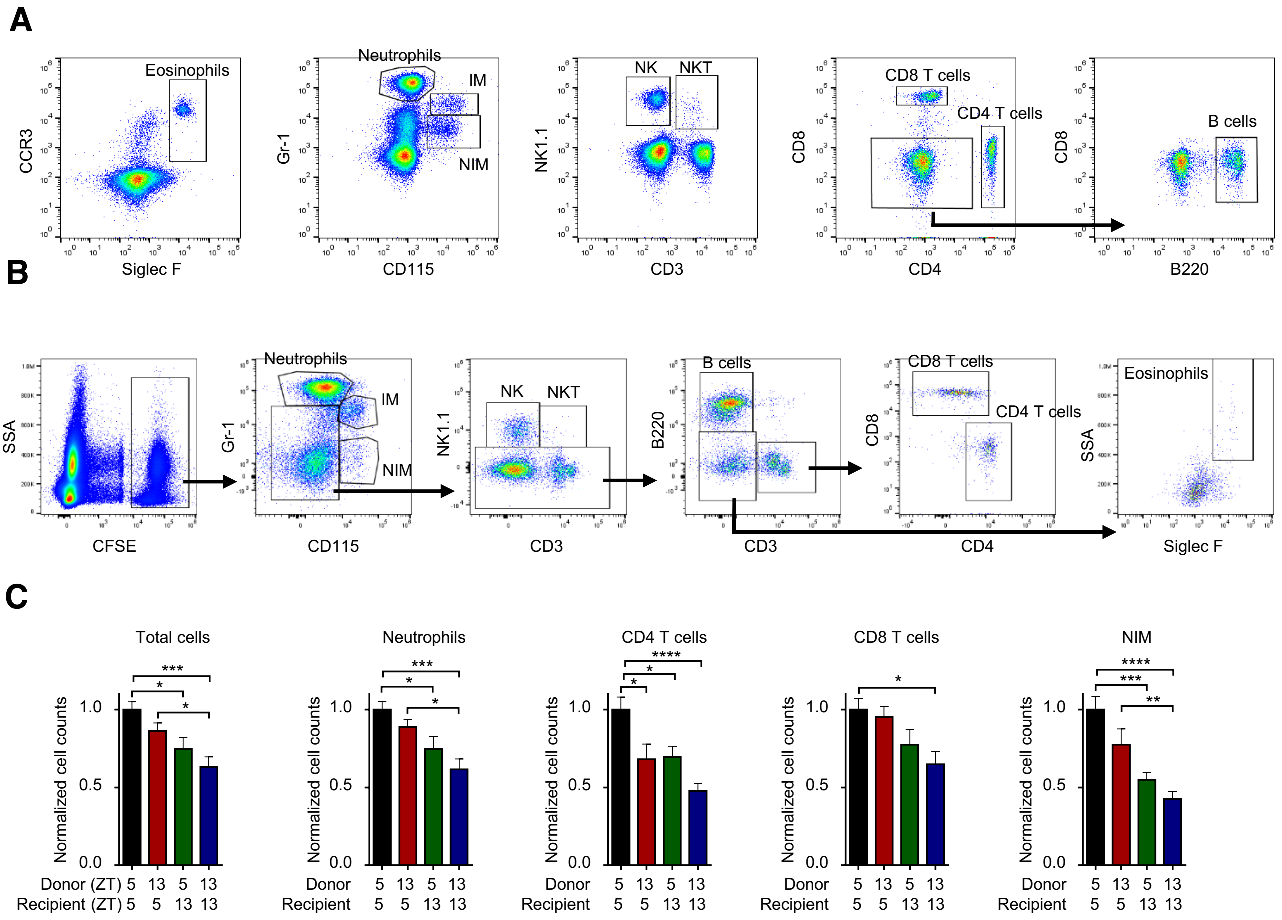

Figure S1 (Related to Figure 1) *Gating strategies and reciprocal homing assays*

(A) Gating strategy of blood leukocyte subsets for Figure 1A. **(B)** Gating strategy for identifying adoptively transferred CFSE$^+$ leukocyte subsets in recipient blood. **(C)** Reciprocal 'negative' homing assays with ZT5 and ZT13 donor cells labeled differently and co-injected into ZT5 and ZT13 recipients; n = 21-24 mice, one-way ANOVA followed by Tukey's multiple comparison test. *p<0.05, **p<0.01, ***p<0.001, ****p<0.0001.

Figure S2

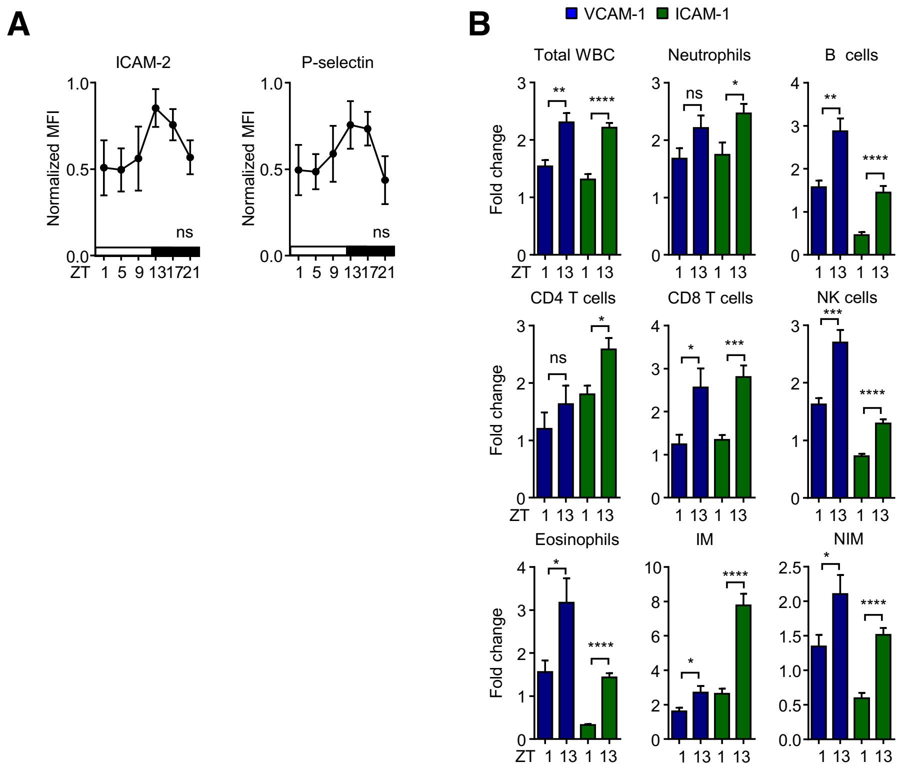

<u>**Figure S2 (Related to Figure 2)** ***Oscillations in endothelial cell adhesion molecules and associated functions***</u>

(A) Integration of ICAM-2 and P-selectin expression over all organs across the day; n = 3-6 mice with 6 time points measured each, one-way ANOVA. **(B)** Fold change of endogenous leukocyte numbers after treatment with antibodies directed against VCAM-1 or ICAM-1 compared to ZT1 and ZT13 isotype-treated control groups; n = 7-11 mice, unpaired Student’s t-test. *p<0.05, **p<0.01, ***p<0.001, ****p<0.0001. ns, not significant.

Figure S3

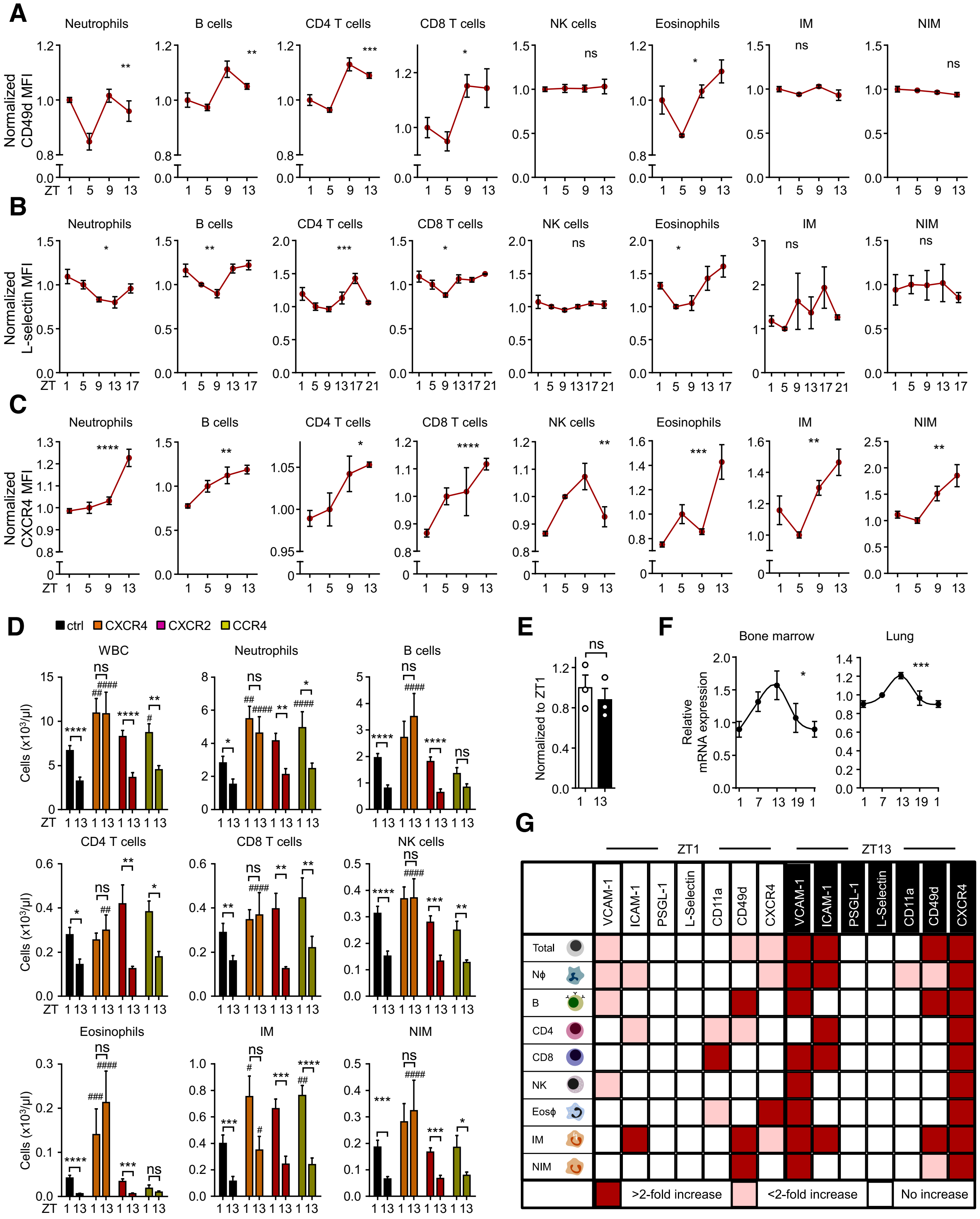

A
Neutrophils
B cells
CD4 T cells
CD8 T cells
NK cells
Eosinophils
IM
NIM
Normalized CD49d MFI
ZT
B
Normalized L-selectin MFI
C
Normalized CXCR4 MFI
D
ctrl
CXCR4
CXCR2
CCR4
WBC
Cells (x10³/µl)
E
Normalized to ZT1
F
Bone marrow
Lung
Relative mRNA expression
G
ZT1
ZT13
VCAM-1
ICAM-1
PSGL-1
L-Selectin
CD11a
CD49d
CXCR4
Total
Nϕ
B
CD4
CD8
NK
Eosϕ
IM
NIM
>2-fold increase
<2-fold increase
No increase

Figure S3 (Related to Figure 3) ***Oscillations in leukocyte pro-migratory factors and associated functions***

(A-C) Expression levels of CD49d **(A)**, L-selectin **(B)** and CXCR4 **(C)** on leukocyte subsets over 24h; n = 3-6 mice with 4-6 time points measured each, one-way ANOVA. **(D)** Endogenous blood leukocyte numbers after treatment with chemokine receptor antagonists; n = 5-12 mice, one-way ANOVA followed by Dunnett comparison to control groups. **(E)** Adoptive transfer of donor cells treated *ex vivo* prior to transfer to recipients with antagonists against CXCR4 at ZT1 and ZT13. Data are normalized to ZT1 levels; n = 3 mice, unpaired Student's t-test. **(F)** *Cxcl12* mRNA levels in bone marrow and lung, ZT1 is double plotted to facilitate viewing; n = 4-5 mice, one-way ANOVA. **(G)** Overview of functional blocking effects on endogenous leukocyte subsets in blood targeting the indicated molecules at ZT1 and ZT13; n = 4-12 mice, one-way ANOVA followed by Dunnett comparison to control groups. *p<0.05, **p<0.01, ***p<0.001, ****p<0.0001; $^{\#, \#\#, \#\#\#, \#\#\#\#}$ indicate analogous significance levels to controls. ns, not significant.

Figure S4

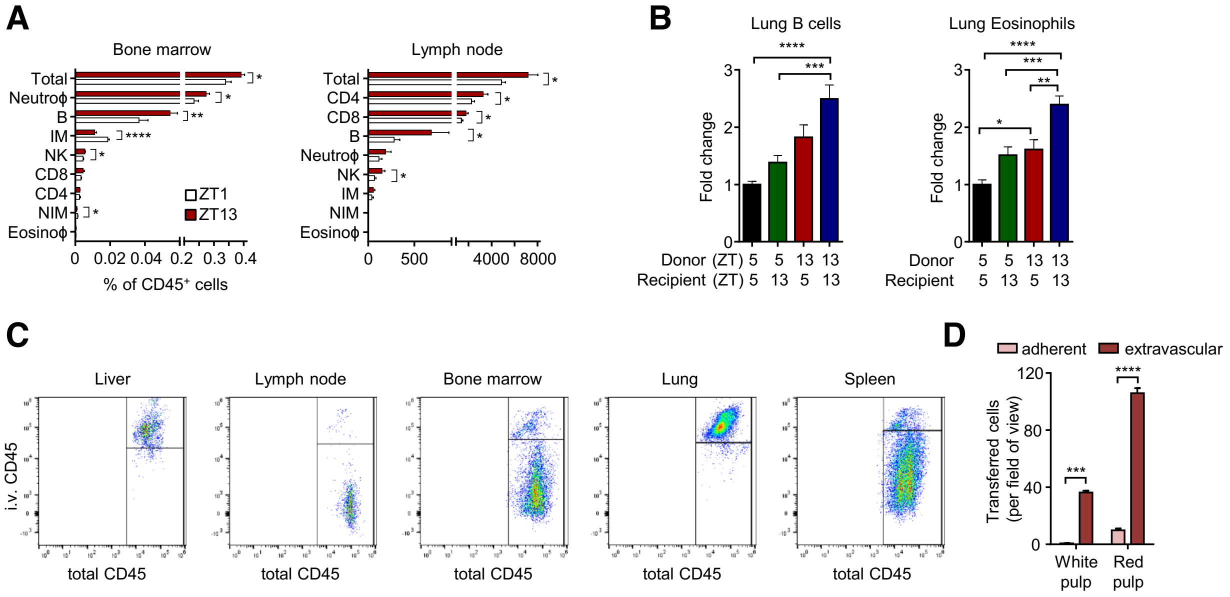

Figure S4 (Related to Figure 4) ***Diurnal homing capacity of leukocyte subsets and localization in specific organs***

(A) Recruitment of donor cells into bone marrow and lymph nodes with ZT1 and ZT13 donor cells and recipients; bone marrow, n = 24-25 mice; lymph node, n = 11-14 mice, unpaired Student's t-test. **(B)** Reciprocal homing assays with ZT5 and ZT13 donor cells labeled differently and co-injected into ZT5 and ZT13 recipients; n = 10 mice, one-way ANOVA followed by Tukey's multiple comparison test. **(C)** Flow cytometry plots of adoptively transferred cells located inside or outside the vasculature in specific organs based on staining with an i.v.-injected anti-CD45 antibody. **(D)** Quantification of numbers and localization of adoptively transferred cells in splenic white pulp and red pulp; n = 2-3 mice, unpaired Student's t-test. *p<0.05, **p<0.01, ***p<0.001, ****p<0.0001.

Figure S5

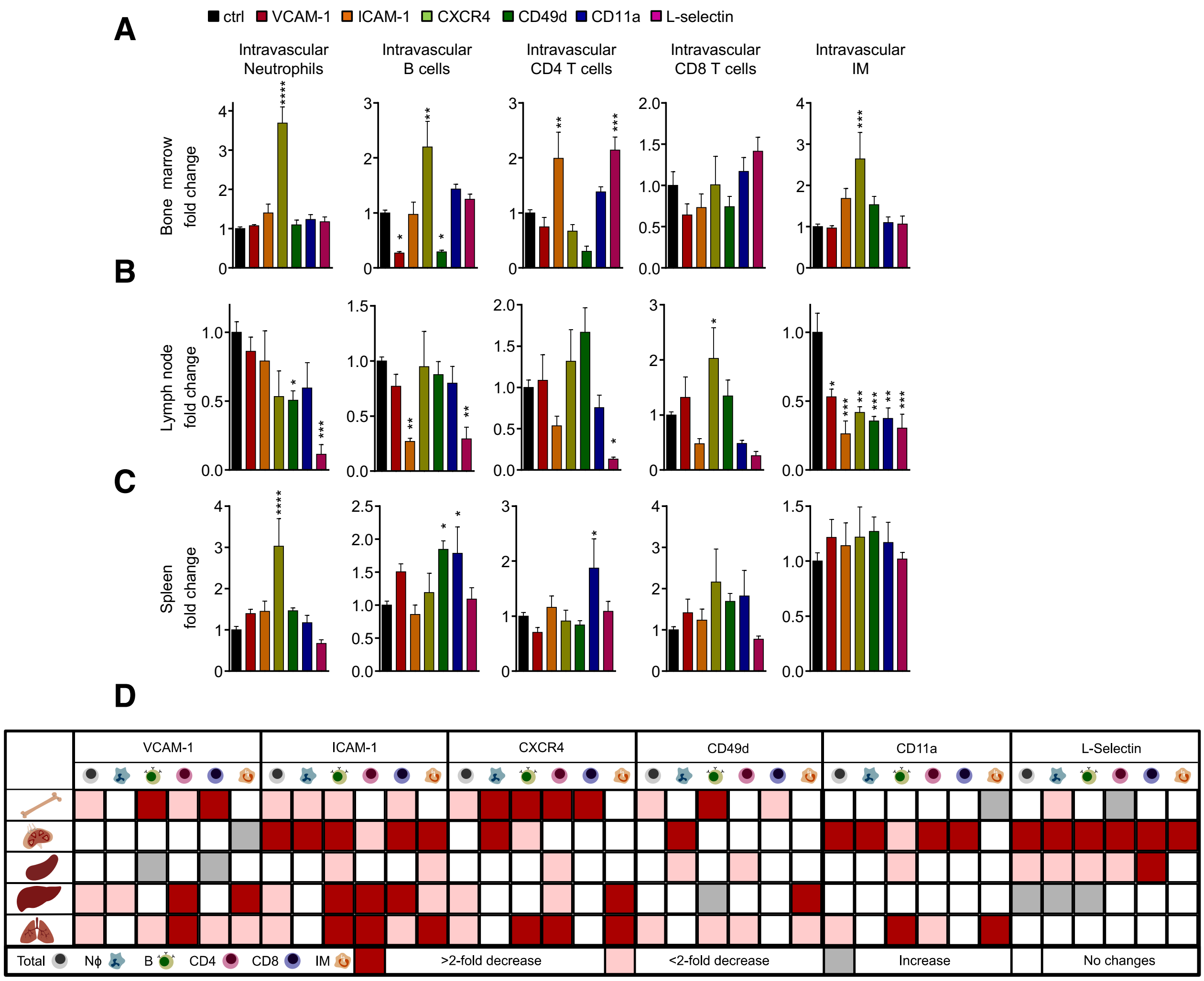

<u>Figure S5 (Related to Figure 5) *Chronopharmacological targeting of leukocyte homing to tissues*</u>

(A-C) Numbers of intravascular donor cells after adoptive transfer and treatment with antibodies or antagonists directed against the indicated molecules, n = 4-8 mice, one-way ANOVA followed by Dunnett comparison to the control group. **(D)** Overview of functional blocking effects on leukocyte recruitment to organs targeting the indicated molecules, n = 4-8 mice, one-way ANOVA followed by Dunnett comparison to the control group. *p<0.05, **p<0.01, ***p<0.001, ****p<0.0001.

Figure S6

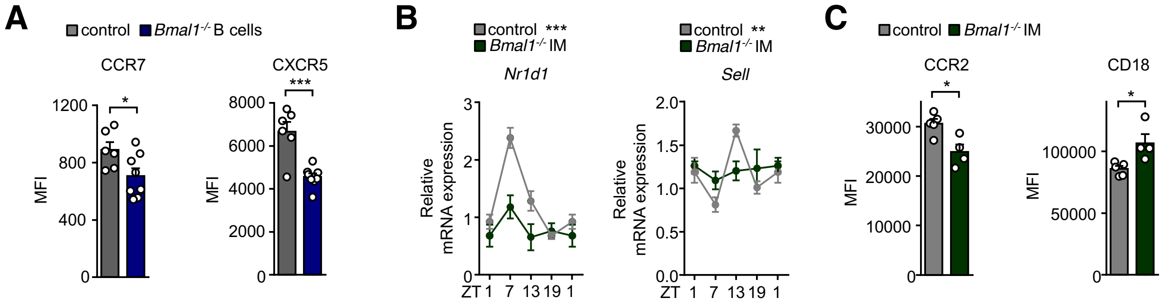

<u>Figure S6 (Related to Figure 6) *Expression of leukocyte adhesion molecules in lineage-specific Bmal1$^{-/-}$ leukocytes*</u>

(A) Expression levels of CCR7 and CXCR5 at ZT13 in control and *Bmal1*-deficient B cells in blood; n = 6-8 mice, unpaired Student's t-test. **(B)** Q-PCR analyses of *Nr1d1* and *Sell* mRNA levels in isolated control and *Bmal1*-deficient monocytes; n = 3 mice, one-way ANOVA. **(C)** Expression levels of CCR2 and CD18 at ZT13 in control and *Bmal1*-deficient monocytes in blood; n = 4-5 mice, unpaired Student's t-test. *p<0.05, **p<0.01, ***p<0.001, ****p<0.0001.

Figure S7

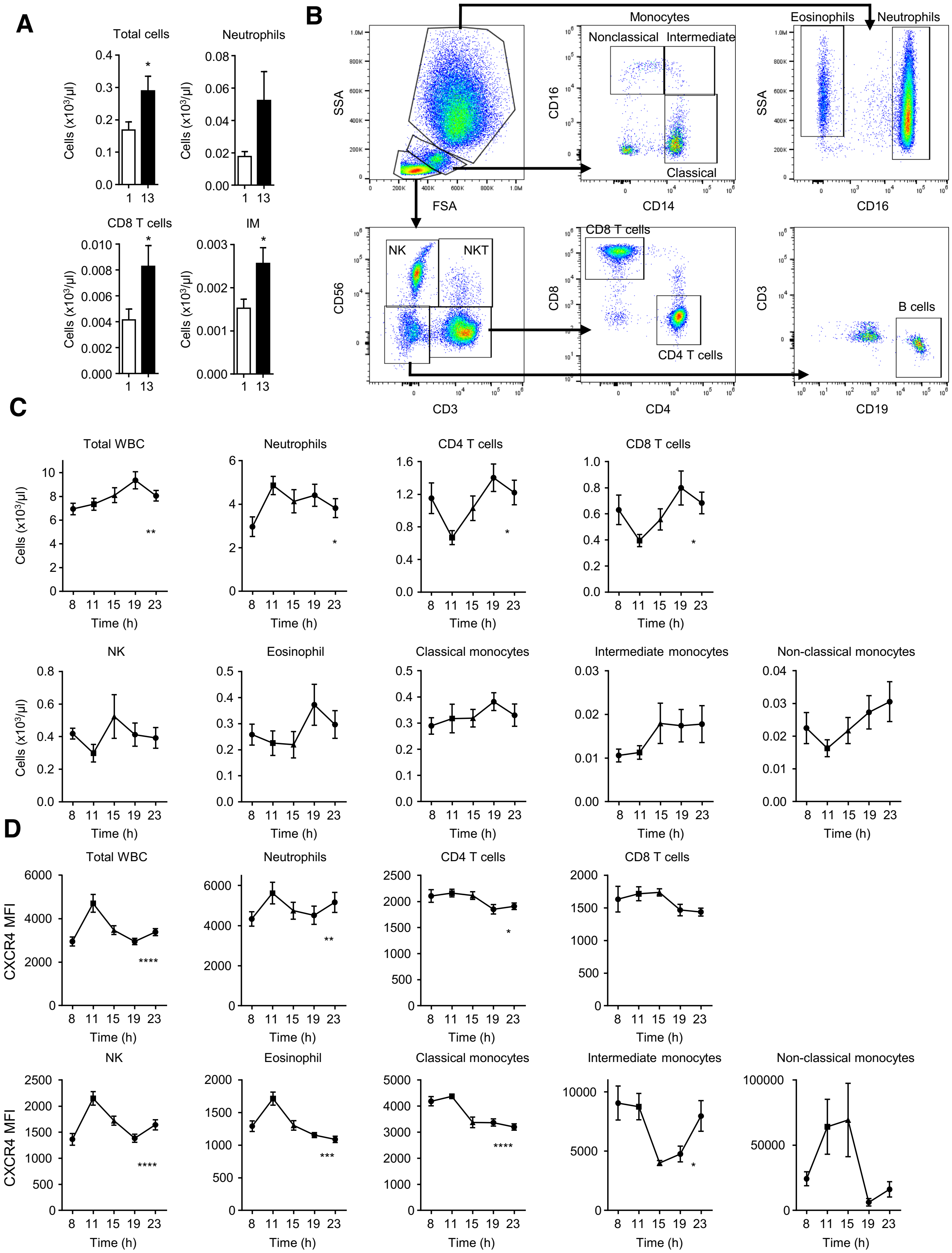
A
Total cells
Neutrophils
CD8 T cells
IM
Cells (x10³/µl)
1
13
B
SSA
FSA
Monocytes
Nonclassical
Intermediate
Classical
CD16
CD14
Eosinophils
Neutrophils
NK
NKT
CD56
CD3
CD8 T cells
CD4 T cells
CD8
CD4
B cells
CD19
C
Total WBC
Neutrophils
CD4 T cells
CD8 T cells
NK
Eosinophil
Classical monocytes
Intermediate monocytes
Non-classical monocytes
Time (h)
D
CXCR4 MFI

Figure S7 (Related to Figure 7) *Oscillations in inflammation and human leukocytes in blood*

(A) Numbers of total leukocytes and subsets in the peritoneal cavity after i.p. LPS challenge at ZT1 and ZT13; n = 12-14 mice, unpaired Student's t-test. **(B)** Gating strategy of human blood leukocyte subsets. **(C)** Numbers of human blood leukocyte subsets over 24h; n = 8 subjects, repeated measures one-way ANOVA. **(D)** Expression of CXCR4 on human leukocytes over 24h; n = 8 subjects, repeated measures one-way ANOVA. *p<0.05, **p<0.01, ***p<0.001, ****p<0.0001.
